# Metabolic and taxonomic insights into the Gram-negative natural rubber degrading bacterium *Steroidobacter cummioxidans* sp. nov., strain 35Y

**DOI:** 10.1371/journal.pone.0197448

**Published:** 2018-05-31

**Authors:** Vikas Sharma, Gabriele Siedenburg, Jakob Birke, Fauzul Mobeen, Dieter Jendrossek, Tulika Prakash

**Affiliations:** 1 School of Basic Sciences, Indian Institute of Technology (IIT) Mandi, Mandi, India; 2 Institute of Microbiology, University of Stuttgart, Stuttgart, Germany; University of Münster, GERMANY

## Abstract

The pathway of rubber (poly [*cis*-1,4-isoprene]) catabolism is well documented for Gram-positive rubber degraders but only little information exists for Gram-negative species. The first documented potent rubber degrading Gram-negative strain is *Xanthomonas* sp. strain 35Y that uses extracellular rubber oxygenases for the initial cleavage of the polyisoprene molecule. However, neither the exact phylogenetic position of *Xanthomonas* sp. strain 35Y nor the catabolic pathway of the primary polyisoprene cleavage products have been investigated. In this contribution, we started to address both these issues by a comprehensive taxonomic characterization and by the analysis of the draft genome sequence of strain 35Y. Evaluation of the 16S rRNA gene sequence pointed to a borderline taxonomic position of strain 35Y as a novel species of the genus *Steroidobacter*. Further, substantial differences in the genotypic properties of strain 35Y and the members of the genus *Steroidobacter*, including average nucleotide identity (ANI) and *in silico* DNA-DNA hybridization (DDH), resolved the taxonomic position of strain 35Y and suggested its positioning as a novel species of the genus *Steroidobacter*. This was further confirmed by comparative analysis of physiological and biochemical features of strain 35Y with other members of the genus *Steroidobacter*. Thus, we conclude that strain 35Y represents a novel species of the genus *Steroidobacter*, for which we propose the designation *Steroidobacter cummioxidans* sp. nov., strain 35Y^T^. A comprehensive analysis of the draft genome of *S*. *cummioxidans* strain 35Y revealed similarities but also substantial differences to rubber degrading Gram-positive counterparts. In particular, the putative transporters for the uptake of polyisoprene cleavage products differ from Gram-positive rubber degrading species. The draft genome sequence of *S*. *cummioxidans* strain 35Y will be useful for researchers to experimentally verify the predicted similarities and differences in the pathways of polyisoprene catabolism in Gram-positive and Gram-negative rubber degrading species.

## Introduction

Natural rubber (NR) is a hydrocarbon biopolymer of *cis*-1,4-isoprene and is widely used in many applications in household and industry. In 2015, the world production of rubber has been raised to 26.7 million tons (46% NR and 54% synthetic rubber) [1]. This huge use of NR for more than a century and the disposal of rubber waste materials in the environment has become a serious problem in all parts of the world. For example, the release of NR or its derivatives as effluents from NR-processing industries causes pollution of rivers and other aqueous ecosystems. The conventional NR waste-management strategies, including landfills and incineration are themselves a large source of environmental pollution. NR-recycling processes, such as pyrolysis and disposal of rubber wastes to the air, water, and soil lead to permanent pollution of the environment. NR-processing can also cause health hazards, including asthma and allergy [2]. All these unwanted consequences of the use of rubber rationalize the necessity for an efficient, economic, and eco-friendly process of NR waste-management.

Bio-remediation is a potential and an emerging alternative for the conventional waste-management strategies. A few microbes are already known for their ability to bio-degrade NR. Most of the known NR-degrading bacteria are Gram-positive *Actinobacteria* [3], including *Streptomyces* sp. K30 [4–6], *Gordonia polyisoprenivorans* VH2 [7], *Nocardia nova* SH22a [8, 9], and *Rhodococcus rhodochrous* RPK1 [10]. They synthesize a latex clearing protein (Lcp), which is the key enzyme in rubber degrading Gram-positive bacteria catalyzing the oxidative cleavage of polyisoprene double bonds. The typical incubation period of these bacteria under laboratory conditions on rubber-latex varies from 6 to 12 weeks [11], thus demonstrating a slow growth with polyisoprene as a sole carbon-source. The complete NR-degradation pathway has been well-characterised in Gram-positive bacteria [7, 9].

Only a few Gram-negative NR-degrading bacteria have been isolated and are described in the literature so far, the first being *Xanthomonas* sp. strain 35Y [12] and recently *Rhizobacter gummiphilus* NS21 [13, 14]. *Xanthomonas* sp. strain 35Y is able to cause a ~60% weight loss of solid NR pieces within one week of incubation [12], thus identifying this strain as one of the relatively faster NR-degrading microbes in comparison to other described NR-degraders. The initial cleavage of poly(*cis*-1,4-isoprene) by *Xanthomonas* sp. strain 35Y is catalyzed by two cooperatively acting extracellular rubber oxygenase enzymes (RoxA and RoxB) which cleave NR to a C_15_ compound, 12-oxo-4,8-dimethyl-trideca-4,8-diene-1-al (ODTD) as a major cleavage product [15, 16]. RoxA and RoxB of *Xanthomonas* sp. strain 35Y are both dihaem cytochrome *c* dioxygenase proteins whereas Lcps are *b*-type cytochromes. RoxAs and Lcps have been thoroughly investigated in the last decade [17, 18, 19, 20, 21], while RoxB has been discovered only recently [16]. The rubber-cleaving system of *R*. *gummiphilus* NS21 [13, 14] is similar to that of *Xanthomonas* sp. strain 35Y and also consists of two rubber oxygenases (RoxA and RoxB) (data unpublished). RoxA and RoxB (alternative designation of RoxB is LatA) of *R*. *gummiphilus* NS21 are highly similar in amino acid sequences to the respective RoxA and RoxB orthologs in *Xanthomonas* sp. strain 35Y. However, no significant similarity has been found between the sequences of RoxAs or RoxBs and Lcps. Nevertheless, RoxA, RoxB, and Lcp are all haem-dependent rubber oxygenases and catalyze an analogous reaction, i.e. the cleavage of NR. The complete NR-degradation process in Gram-negative bacteria remains unknown.

Currently, the application of NR-bioremediation is impeded due to the lack of a comprehensive understanding of the mechanistic details of the microbial NR-degradation process at the metabolic level. To unravel the complete NR-degradation pathway in Gram-negative rubber degraders, *Xanthomonas* sp. strain 35Y is an important candidate. However, the unavailability of its genome sequence has so far been a major hurdle in exploring its metabolic potentials. In addition, the exact taxonomic position of *Xanthomonas* sp. strain 35Y is obscure. The first and only publication describing the source and taxonomy of *Xanthomonas* sp. strain 35Y [12] states that *Xanthomonas* sp. strain 35Y was taken from the culture collection of the authors’ institute. A comprehensive taxonomic analysis was not performed for *Xanthomonas* sp. strain 35Y, except for the statement of a strict respiratory metabolism, the identification of the Gram status, the identification of an insoluble yellow pigment, and the presence of one or two flagella. From these data, strain 35Y was tentatively designated as *Xanthomonas* sp. strain 35Y. The detailed description of strain 35Y, that was announced in the publication from 1990 [12], to the best of our knowledge was never published.

The unavailability of the genome sequence and the unclear taxonomic position, but the otherwise great importance of *Xanthomonas* sp. strain 35Y for the understanding of the catabolism of rubber in Gram-negative bacteria, prompted us to perform the present study. To this end, we sequenced and annotated the draft genome of *Xanthomonas* sp. strain 35Y and performed a comprehensive analysis to gain insights into its general genomic features and metabolic potential with special emphasis on putative proteins involved in the catabolism of NR. Furthermore, we determined and evaluated several physiological and biochemical characteristics in combination with the analysis of the draft genome sequence which allowed us to perform a reliable taxonomic characterization of strain 35Y and to identify strain 35Y as a novel species of the genus *Steroidobacter*, for which we propose the designation *Steroidobacter cummioxidans* sp. nov., strain 35Y^T^.

## Materials and methods

### Bacterial strains

*S*. *cummioxidans* strain 35Y was first described by Tsuchii and Takeda in 1990 [12] as *Xanthomonas* sp. strain 35Y and was obtained from their laboratory. It has been deposited to Leibniz Institute Deutsche Sammlung von Mikroorganismen und Zellkulturen (DSMZ) Germany (DSM 103114). The genome and protein sequences of other bacterial strains used for comparative genomics in this study **(Table A in S1 Text)** were retrieved from the GenBank database (http://www.ncbi.nlm.nih.gov/genbank/).

### Bacterial growth

*S*. *cummioxidans* strain 35Y was grown in lysogeny broth (LB, 10 g tryptone, 10 g NaCl and 5 g yeast extract per liter) at 30°C, 100 rpm for 48 h.

### DNA extraction

20 ml of *S*. *cummioxidans* strain 35Y culture was grown in LB medium (30°C, 100 rpm). The cells were centrifuged (10 minutes, 5000 *g*, 4°C) and re-suspended in potassium phosphate buffer (100 mM, pH 7.0). After a second centrifugation, cells were re-suspended in 1.7 ml saccharose-Tris (saccharose: 25 g; Tris 0.12 g; pH 8.0; 100 ml water) followed by the addition of 200 μl lysozyme solution (stock: 20 mg/ml). Then, 2.5 ml saccharose-Tris, 300 μl proteinase K solutions (2.5 mg/ml) and 2.5 ml sodium dodecyl sulfate solution (0.15 g in 100 ml water) were added and incubated for 2.5 h at 37°C. Subsequently, 1.88 ml sodium perchlorate solution (NaClO_4_: 70.32 g; water: 100 ml; acetic acid: pH 4.8) was added and mixed gently, then 2.5 ml phenol-chloroform-isoamylalcohol (25:24:1) was added and mixed gently. After centrifugation (10 minutes, 5000 *g*, 4°C), the aqueous phase was used for a second phenol-chloroform-isoamylalcohol extraction and subsequently, an extraction with 2.5 ml chloroform-isoamylalcohol (24:1). The aqueous phase was transferred into a new falcon tube, mixed with 25 μl RNAse solution (10 mg/ml) and incubated for 30 minutes at 37°C. The genomic DNA was extracted with 1.5 volumes of isopropanol and washed 3x5 minutes with 70% ethanol. The DNA was dried at 60°C and re-suspended in 2 ml water.

### Genome sequencing and assembly

Genomic shotgun libraries were sequenced by the Goettingen Genomics Laboratory (G2L, http://www.appmibio.uni-goettingen.de/index.php?sec=g2l/) by a combination of 454 pyrosequencing and Illumina sequencing technologies. The 454 shotgun library was generated using the GS FLX Rapid Library Prep Kit (454 Roche, Branford) and the genome was sequenced on the 454 FLX instrument (454 Roche, Branford) using XL+ chemistry. The shotgun library for Illumina sequencing was done by Nextera DNA Sample preparation kit (Illumina, San Diego, USA) following the manufacturer’s instructions and the genome was sequenced on the Illumina GA IIx instrument using a 112 bp paired end single indexed run technology. The number of reads generated by the 454 pyrosequencing and Illumina sequencing technologies were 101,250 and 6,494,354, respectively. Hybrid genome assembly was done using default parameters of the MIRA (Mimicking Intelligent Read Assembly) assembler (version as of February, 2013) [22] which resulted in 127 contigs in total, exhibiting an average coverage of 34.47X. Upon screening of the resultant assembly for contaminations, including PhiX sequences using BLASTN (version 2.2.29+), 126 contigs were obtained.

### Basic genome analysis

Open reading frames (ORFs) and proteins of 126 contigs were predicted by IMG/ER (http://www.img.jgi.doe.gov/cgi-bin/mer/main.cgi/). ORFs were annotated using BLASTP (version 2.2.29+) against the NCBI NR protein database (version as of October 20, 2014) with a bit-score of 50 and an e-value cut-off of 10^−6^. All contigs were used for the prediction of tRNA and rRNA genes using tRNA and hmm_rRNA prediction modules of WebMGA server (http://weizhong-lab.ucsd.edu/metagenomic-analysis/). For comparative analysis, assignment of orthologs, and similarity searches, a threshold of 30% amino acid sequence identity and 60% coverage of both query and hit in the alignment were used. In order to find orthologs, predicted protein sequences of *S*. *cummioxidans* strain 35Y were blasted (BLASTP version 2.2.29+) against bacterial strains used in this study **(Table A in S1 Text)** using an e-value cut-off of 10^−4^ and the best hits were selected.

Functional annotation of all predicted protein sequences was done using Clusters of Orthologous Groups (COGs) module of WebMGA server (http://hweizhong-lab.ucsd.edu/metagenomic-analysis/server/cog/) with an e-value cut-off of 10^−4^. Pathway mapping was done using BlastKOALA module of Kyoto Encyclopedia of Genes and Genomes (KEGG) online server (http://www.kegg.jp/blastkoala/) using prokaryotes as the taxonomy group and genus_prokaryotes as the KEGG genes database. PSORTb v3.0.2 server (http://www.psort.org/psortb/) was used for the prediction of the subcellular-localization (parameters used, organism: bacteria; Gram-stain: negative). SecretomeP v2.0 was used for the prediction of non-classically secreted proteins (http://www.cbs.dtu.dk/services/SecretomeP/). Transporter proteins were predicted using Transporter Classification Database (TCDB) database (http://www.tcdb.org/, version as of March 25, 2015) by performing BLASTP (version 2.2.29+) with an e-value cut-off of 10^−10^. To predict the carbohydrate active enzymes, the standalone version of Database for Automated Carbohydrate-Active Enzyme Annotation (dbCAN HMMs v5.0) (http://csbl.bmb.uga.edu/dbCAN/) was used. The results were parsed with the following two parameters recommended for bacteria (i) if alignment >80 amino acids, use an e-value < 10^−18^, otherwise use e-value < 10^−3^ and (ii) covered fraction of HMM > 0.35. Promoter sequences were predicted on 126 contigs using Neural Network Promoter Prediction (NNPP) v2.2 server (http://www.fruitfly.org/seq_tools/promoter.html/) using a minimum promoter score of 0.8 and, including reverse strands.

MvirDB database (http://www.mvirdb.llnl.gov/, version as of February 19, 2015) was used for predicting the virulence factors using BLASTP (version 2.2.29+) with an e-value cut-off of 10^−10^. To predict the antibiotic resistant genes, Resistance Gene Identifier (RGI) module of Comprehensive Antibiotic Resistance Database (CARD) database (https://card.mcmaster.ca/) was used with default parameters. To predict secreted proteins and protein secretion systems, EffectiveDB server (http://www.effectivedb.org/) was used with enabled “genome mode” and prediction module “EffectiveS346”. PHAge Search Tool (PHAST) (http://phast.wishartlab.com/) and Phage Classification Tool Set (PHACTS) (http://www.phantome.org/PHACTS) servers were used on 126 contigs of *S*. *cummioxidans* strain 35Y for the prediction of prophages. ISfinder server (https://www-is.biotoul.fr/) was used with default parameters for the prediction of insertion elements. CRISPRfinder server (http://crispr.i2bc.paris-saclay.fr/Server/) was used with default parameters to identify the putative clustered regularly interspaced short palindromic repeats (CRISPRs).

### *In silico* analysis of rubber oxygenases

For the functional analysis of rubber oxygenases and their homologs by classifying them into families and predicting domains and important sites, InterPro (https://www.ebi.ac.uk/interpro/) server was used.

### Taxonomy analysis of strain 35Y

The taxonomic positioning of strain 35Y was determined using polyphasic taxonomic approaches, including (i) 16S rRNA gene based method, (ii) average nucleotide identity (ANI) based method, (iii) *in silico* DNA-DNA hybridization (DDH) estimate based method, and (iv) physiological and biochemical characterization.

### (i) 16S rRNA gene based method

The 16S rRNA gene sequence of strain 35Y (STC_rRNA4) was used for online BLASTN (version 2.8.0+) analysis against the 16S ribosomal RNA sequences (Bacteria and Archaea) database of NCBI webserver (https://blast.ncbi.nlm.nih.gov/Blast.cgi?PAGE_TYPE=BlastSearch, version as of February 05, 2018) which revealed high identity with members of the class *Gammaproteobacteria*. For further analysis, 16S rRNA gene sequence from at least one member from each genus (except for unclassified groups and environment samples) under the class *Gammaproteobacteria* was taken for the phylogenetic study. The 16S rRNA gene sequence of strain 35Y was showing highest identity with that of the genus *Steroidobacter* under the family *Sinobacteraceae*, hence all the available members of this family were used for this analysis. Evolutionary analyses were conducted in Molecular Evolutionary Genetics Analysis 6 (MEGA6) [23] software. The sequences were aligned using ClustalW module of MEGA6 with default parameters. The evolutionary history was inferred by using the maximum likelihood method based on the Kimura 2-parameter model [24]. Robustness of the phylogenetic trees was evaluated using the bootstrap resampling method of Felsenstein [25]. The bootstrap consensus tree inferred from 1000 replicates is taken to represent the evolutionary history of the taxa analyzed. Initial trees for the heuristic search were obtained automatically by applying neighbor-join and BioNJ algorithms to a matrix of pairwise distances estimated using the maximum composite likelihood approach and then selecting the topology with superior log likelihood value. *Nitrosomonas aestuarii* Nm69 was used as the outgroup. The tree was drawn to scale, with branch lengths measured in the number of substitutions per site. The analysis involved 58 nucleotide sequences. All positions containing gaps and missing data were eliminated. There were a total of 1,146 positions in the final dataset. In addition, BLASTN (version 2.2.29+) was used to calculate the percent identity between the 16S rRNA gene sequences for a given pair of bacteria. FigTree v1.4.2 [26] was used for tree-visualization.

### (ii) Average nucleotide identity based method

The complete and draft quality genomes of *S*. *denitrifican*s DSM 18526 and strain 35Y, respectively were used for the calculation of the ANI value [27]. Towards this, ANIb module of standalone version of JSpecies v1.2.1 [28] was used with default parameters.

### (iii) *In silico* DNA-DNA hybridization estimate based method

Genome-to-Genome Distance Calculator (GGDC) v2.1 server (http://ggdc.dsmz.de/distcalc2.php) was used to calculate genome to genome distance of strain 35Y with respect to *S*. *denitrifican*s DSM 18526 in order to determine *in silico* DDH estimate.

### (iv) Physiological and biochemical characterization

A comprehensive literature survey was carried out for the physiological and biochemical characterization of strain 35Y.

### Detection of polyhydroxyalkanoate granules

Morphological analysis and fluorescence microscopy were performed on a Nikon Ti-E microscope (MEA53100) equipped with a Hamamatsu Orca Flash 4.0 sCMOS camera. The formation of polyhydroxyalkanoate (PHA) granules was followed after staining the cells (grown on basal broth medium supplemented with glucose) with Nile red [29] (1–10 μg/ml in DMSO or ethanol) and a filter set with extinction at 562/40 nm and emission at 594 LP. To further validate our results, the gas chromatography analysis was carried out after acidic methanolysis of lyophilized cells under PHA permissive cultivation conditions as per standard protocol [30].

### Detection of polyphosphate granules

Polyphosphate (polyP) granules of bacterial cells (grown on nutrient broth medium supplemented with glucose) were stained with 4’,6-diamino-2-phenylindole 2HCl (DAPI) [31] (DAPI, concentration 60 μg/ml) and detected with the aid of a DAPI-polyP specific filter set (ex: 415/20 nm, em: 520/60 nm). *Ralstonia eutropha* H16 cells were used as control. This species is well known for its ability to synthesize both PHA and polyP granules.

## Results and discussion

### Determination of the taxonomic position of *Xanthomonas* sp. strain 35Y

**Fig 1:**
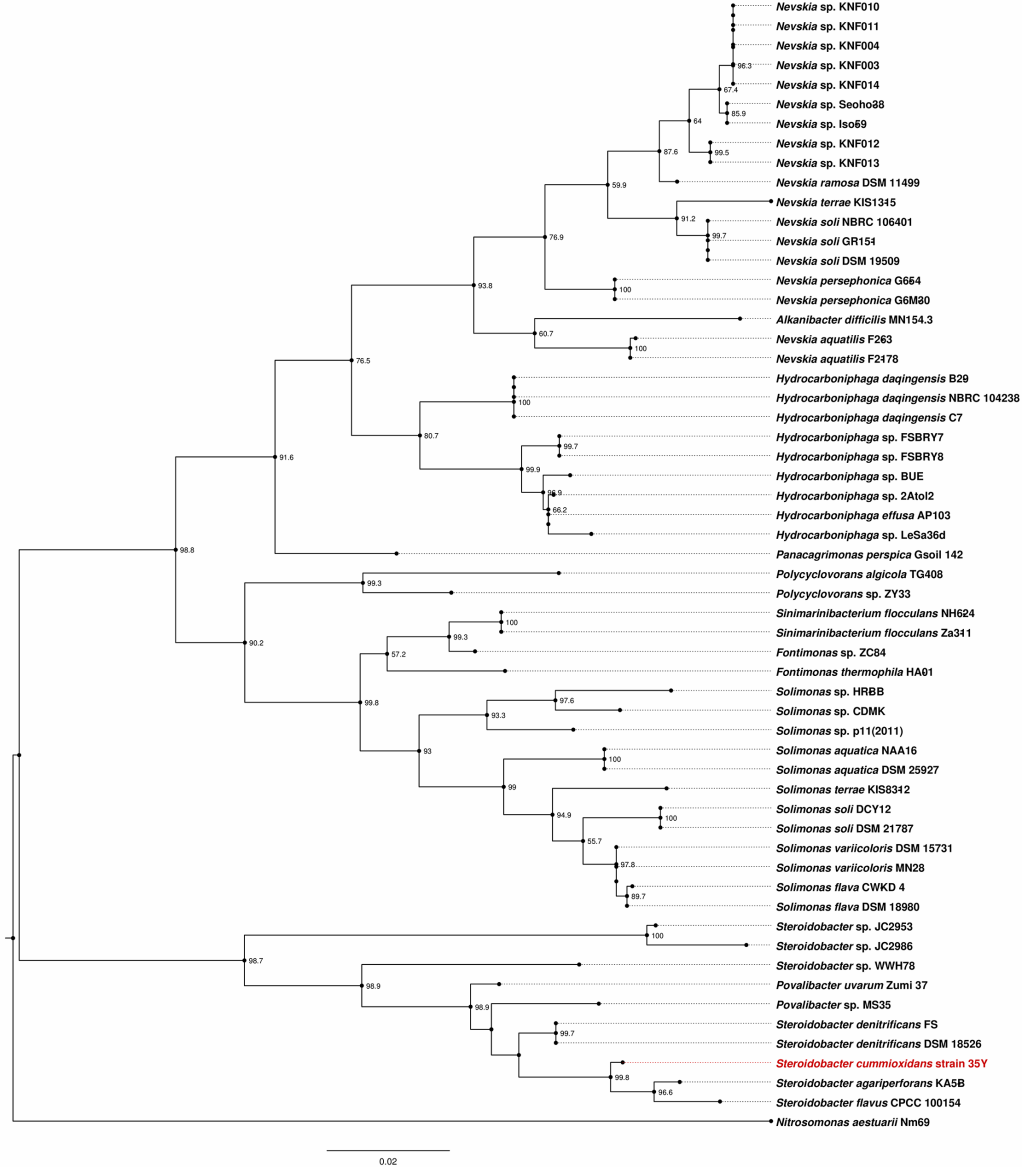
Phylogenetic relationships between *Steroidobacter cummioxidans* sp. nov., strain 35Y and members of the family *Sinobacteraceae* on the basis of 16S rRNA gene sequences. The phylogenetic tree was constructed by using the maximum likelihood method, and the 16S rRNA gene sequence of *Nitrosomonas aestuarii* Nm69 was used as the outgroup. Numbers at nodes indicate levels of bootstrap support (%) based on a maximum likelihood analysis of 1000 resampled datasets; values of less than 50 % are not shown. Bar, 0.02 nucleotide substitutions per site. Solid lines represent the lengths of branches; dotted lines are used to align the tip labels for better visualization; circle represents the node.

The previous taxonomic classification of strain 35Y as a member of the genus *Xanthomonas* was based on the presence of a yellow pigment and strict aerobic respiratory metabolism of the Gram-negative and motile cells of strain 35Y [12]. To determine the taxonomic position of strain 35Y with more reliability, we first performed 16S rRNA gene sequence analysis. The gene sequence of the complete 16S rRNA was a by-product of the determination of the draft genome sequence that is described in detail below. Strain 35Y demonstrated considerably low 16S rRNA gene sequence identities with the members of the genus *Xanthomonas*; the highest value was for *Xanthomonas campestris* ATCC 33913 (85.14%). These data suggest that strain 35Y is not a species of the genus *Xanthomonas*. Furthermore, comparison of the 16S rRNA gene sequence of strain 35Y with the 16S ribosomal RNA sequences (Bacteria and Archaea) database revealed high identities with the members of the genus *Steroidobacter*; the highest value was found for *S*. *flavus* CPCC 100154 (98.27%) followed by *S*. *agariperforans* KA5-B (98.19%) **(Table B in S1 Text)**. The 16S rRNA gene sequence identity with *S*. *denitrificans* DSM 18526^T^, the only member of the genus *Steroidobacter* for which the genome sequence is available, was 97.78%. These data suggest that strain 35Y is a species of the genus *Steroidobacter* within the family *Sinobacteraceae*. The phylogenetic position of strain 35Y in the family *Sinobacteraceae* is shown in **Fig 1**.

In order to delineate the species, Kim *et al*. proposed a threshold of 98.65% 16S rRNA gene sequence identity on a large collection of bacterial species [32]. A 16S rRNA sequence identity of 98.27% with *S*. *flavus* CPCC 100154 presents strain 35Y as a borderline case for species demarcation on the basis of this feature alone. Thus in order to delineate the species of strain 35Y in the genus *Steroidobacter*, we have adopted polyphasic taxonomic approaches. The first approach is based on *in silico* genotypic characterisation and the second approach is based on thorough literature evidence for various physiological and biochemical features.

In the *in silico* based approach, we have used two standard methods of species demarcation, including ANI analysis and *in silico* DDH analysis. An ANI threshold range of 95–96% is proposed to demarcate the species boundary [32, 33, 34]. The ANI value of strain 35Y with *S*. *denitrificans* DSM 18526 was found to be 72.23%, which is too low to consider it as a known species of the genus *Steroidobacter*. The recommended threshold value of species demarcation as per *in silico* DDH based method is 70% [35]. This value was calculated to be 19.8% between strain 35Y and *S*. *denitrificans* DSM 18526 and thus this method also could not classify strain 35Y as a known species of the genus *Steroidobacter*. The genotypic data obtained using three independent approaches, including 16S rRNA gene sequence identity based-, ANI value based-, and *in silico* DDH value based-methods showed that strain 35Y should be considered as a novel species of the genus *Steroidobacter*.

This conclusion was further supported by a comprehensive literature evidence-based evaluation of various physiological and biochemical features of strain 35Y and the other members of the genus *Steroidobacter* **(Table 1)**. As shown in **Table 1,** all members of the genus *Steroidobacter*, including strain 35Y, are non-spore-forming Gram-negative rods. In addition, they are also negative for the hydrolysis of gelatine. Strain 35Y can be distinguished from the close species neighbours by several traits, including being able to hydrolyse casein, growth on complex media, secretion of xanthomonadin pigment, the inability for nitrate-reduction, and being catalase negative. In addition, strain 35Y is also distinguished from the known species of the genus *Steroidobacter* based upon significant differences among their genome sizes and temperature ranges for growth. Taken together, the polyphasic taxonomy analysis indicated strain 35Y as a novel species in the genus *Steroidobacter*. We propose to designate this novel species as *Steroidobacter cummioxidans* to show its rubber oxidizing capacity. Strain 35Y is currently the only member of the species and represents the type strain (35Y^T^).

**Table 1:** Characteristics of *Steroidobacter cummioxidans* strain 35Y (35Y), *Steroidobacter flavus* CPCC 100154 (CPCC 100154), *Steroidobacter agariperforans* KA5-B (KA5-B), *Steroidobacter denitrificansis* DSM 18526 (DSM 18526).

Characteristics	35Y [12, 50, this study]	CPCC 100154 [52]	KA5-B [53]	DSM 18526 [54]
**Flagellar arrangement**	Motile by 1 (or 2) polar flagellum	Motile by single polar flagellum	Non-motile	Motile by single polar flagellum
**Cell size width by length (μm)**	0.4–0.6 by 2.0–5.0	0.6–0.8 by 1.5–1.8	0.4–0.6 by 1.0–2.1	0.3–0.5 by 0.6–1.6
**Cell morphology**	Rod	Rod	Straight to slightly curved rod	Slightly curved rods
**Sporulation**	-	-	-	-
**Gram-stain**	-	-	-	-
**Oxidase**	+	-	+	+
**Catalase**	-	+	+	+
**Utilization as sole carbon source:**				
D-fructose	+	-	+	-
D-maltose	+	-	+	-
Dextrin	+	-	+	-
D-galactose	+	-	+	+
D-rhamnose	ND	-	+	-
D-trehalose	+	+	-	-
D-turanose	+	-	+	-
Tween 40	+	-	+	-
α-D-lactose	+	-	+	-
**Hydrolysis of:**				
Casein	+	-	ND	ND
Gelatine	-	-	ND	ND
Starch	+	-	+	ND
**Anaerobic growth**	-	ND	-	+
**Nitrate reduction**	-	ND	ND	+
**Growth on complex media**	+	ND	ND	-
**Utilization of agar as a C-source**	-	ND	+	-
**Temperature range for growth (°C)**	25–41	20–37	15–37	20–38
**DNA G+C content (mol %)**	60.94	64.4	62.9	61.9
**16S rRNA gene identity (%)**	100	98.28	98.2	97.59
**Genome Size (Mbp)**	7.9	ND	ND	3.5
**Xanthomonadin pigment**	+ C_27_H_28_Br_2_O_2_ [47]	ND	ND	ND
**Xanthomonadin biosynthetic gene cluster [55]**	A cluster of 14 genes	ND	ND	Only 4 genes*

ND: not determined or not available; +: positive; -: negative.

* Details are given in **S1 Text**.

### Description of *Steroidobacter cummioxidans* sp. nov.

*Steroidobacter cummioxidans* sp. nov. (cum.mi.o'xi.dans. L. n. *cummi*, rubber sap; N.L. part. adj. *oxidans*, oxidizing; N.L. masc. adj. *cummioxidans*, oxidizing rubber. The ability to utilize natural rubber (poly [*cis*-1, 4-isoprene]) as a carbon source and to form clearing zones on opaque latex agar is eponymous for the species. The cellular dimensions of *S*. *cummioxidans* strain 35Y are 0.4 to 0.6 μm in width and 2 to 5 μm in length but cells occasionally can become considerably longer (10 μm). The cells are motile rods by one or few polar flagella. The strains of *S*. *cummioxidans* possess a Gram-negative cell wall structure and form yellow-orange colored colonies due to the synthesis of bromine-containing pigments (xanthomonadins). However, because of low 16S rRNA gene identities of *S*. *cummioxidans* strain 35Y^T^ to true *Xanthomonas* species, this strain belongs to the genus *Steroidobacter* despite the formation of xanthomonadins. The strains of *S*. *cummioxidans* rely on a strictly respiratoric metabolism. They are prototrophic and can use a wide range of compounds for growth, including sugars, fatty acids, low molecular weight hydrocarbons, amino acids, and diverse complex media at moderate conditions. Strains of *S*. *cummioxidans* have a temperature range of 25–41°C for growth, thus are mesophilic in nature.

### Basic features of the draft genome of *S*. *cummioxidans* strain 35Y

The draft genome of *S*. *cummioxidans* strain 35Y has a size of almost 8 Mbp (7,936,208 bp) with a DNA G+C content of 60.94 mol% **(Table 2)**. The draft genome has a capacity for 6,906 protein encoding genes as well as 47 and 7 tRNAs and rRNAs, respectively. Further details of the predicted genes and their putative functions are given in **Table 2**, **S1 Dataset and S1 Text**.

**Table 2:** Basic genome features of *Steroidobacter cummioxidans* strain 35Y.

**Number of Contigs**	126 (smallest: 3,181 bp, largest: 336,437 bp, average: ~62,986 bp)		
**Genome Size**	7,936,208 (bp)		
**G+C Content (mol%)**	60.94		
**Number of Genes**	6,960	**Protein encoding genes (PEGs)**	6,906
		**tRNA**	47
		**rRNA**	7
**Proteins with Predicted Functions**	5,475		
**Hypothetical or Uncharacterized Proteins**	1,431		
**Proteins with COG Annotations**	2,637	**Metabolism**	35.61%
		**Cellular processes and signalling**	17.29%
		**Information storage and processing**	15.24%
		**Poorly characterized**	18.54%
		**Multiple classes**	13.31%
**Proteins with KEGG Annotations**	2,892		
**Membrane Transporters**	479		
**Virulence Factors**	909		
**Subcellular Localization**	**Cytoplasmic**	37.85%	
	**Cytoplasmic membrane**	20.50%	
	**Extracellular**	1.51%	
	**Outer membrane**	3.82%	
	**Periplasmic**	2.43%	
	**Unclassified**	33.88%
**Prophages**	2 (incomplete)	10.1 kb	8 PEGs
		9.4 kb	9 PEGs
**Insertion Elements**	90		
**CRISPR Loci**	3		
**Antibiotic Resistant Genes**	50		
**Carbohydrate-Active Enzymes**	269	**Auxiliary Activity**	5.20%
		**Carbohydrate Esterase**	14.50%
		**Carbohydrate-Binding Module**	15.99%
		**Glycoside Hydrolase**	39.03%
		**Glycosyl Transferase**	21.56%
		**Polysaccharide Lyase**	3.72%

### NR-degradation potential of *S*. *cummioxidans* strain 35Y

**Fig 2:**
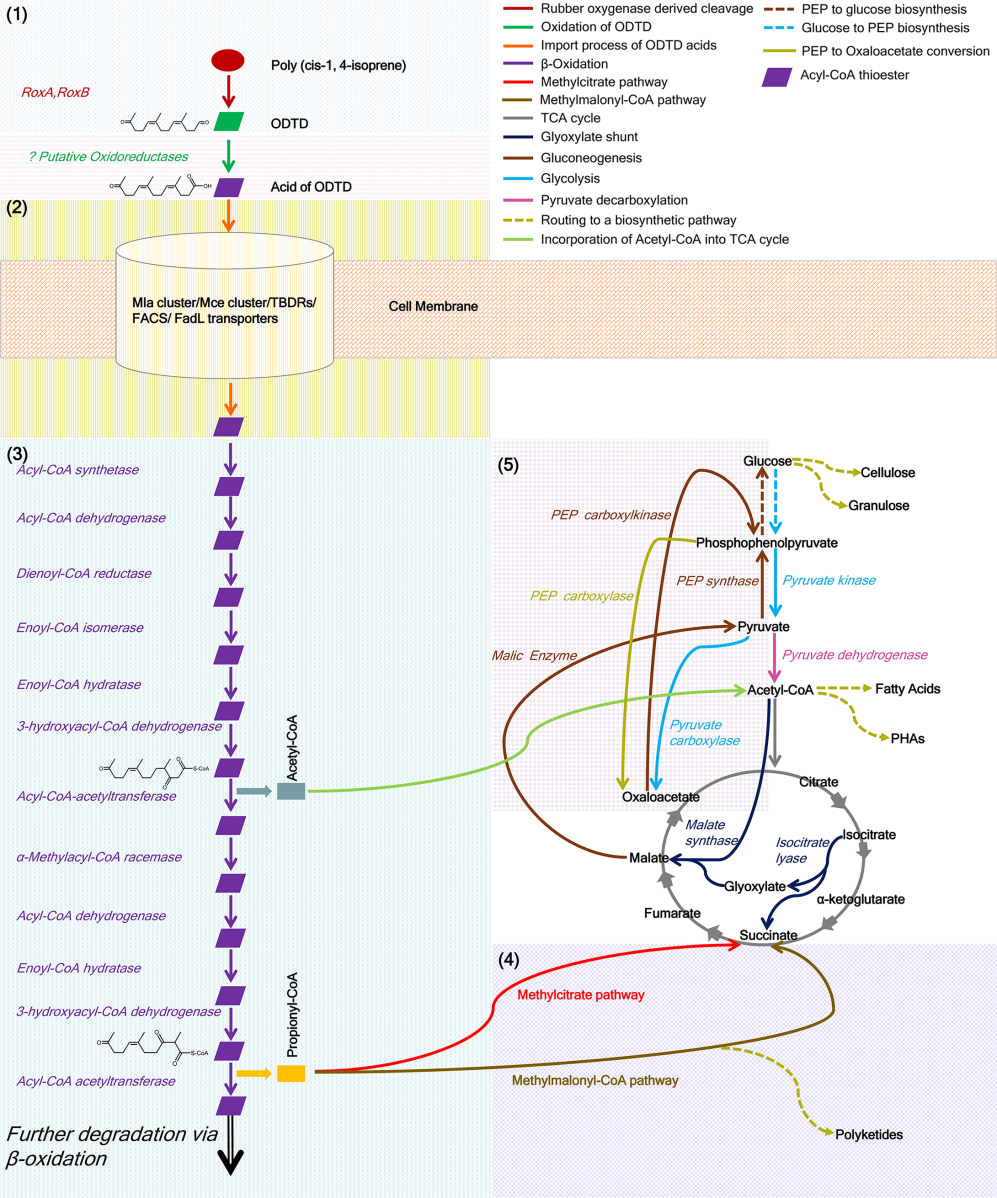
Predicted natural rubber degradation pathway of *Steroidobacter cummioxidans* strain 35Y. The pathway comprises of five steps: (1) biosynthesis of rubber oxygenases and extracellular oxidative cleavage of polyisoprene chains, (2) import of oligoisoprenes, (3) β-oxidation, (4) acetyl-CoA and propionyl-CoA metabolism, and (5) anaplerotic reactions and gluconeogenesis. RoxA: Rubber oxygenase A, RoxB: Rubber oxygenase B, ODTD: 12-oxo-4,8-dimethyltrideca-4,8-diene-1-al, Mce: Mammalian cell entry protein, Mla: An ABC transport system that maintains lipid asymmetry, TBDRs: TonB-dependent outer membrane receptors, FACS: fatty acyl coenzyme A (CoA) synthetase: FadL: It encodes outer membrane proteins/ transporters involved in long-chain fatty acid, TCA: Tricarboxylic acid cycle, PEP: Phosphoenolpyruvate.

NR-degradation related genes were predicted in the draft genome of *S*. *cummioxidans* strain 35Y using comparative analyses with other known NR-degrading bacteria, such as *G*. *polyisoprenivorans* VH2 [7] and *N*. *nova* SH22a [8, 9]. The NR-degradation process can be categorized into five major phases **(Fig 2)**, including (1) biosynthesis of rubber oxygenases and extracellular oxidative cleavage of polyisoprene chains, (2) import of oligoisoprenes, (3) β-oxidation, (4) acetyl-CoA and propionyl-CoA metabolism, and (5) anaplerotic reactions and gluconeogenesis. In the following sections, the main features of the five phases are discussed in detail.

### (1) Biosynthesis of rubber oxygenases and extracellular oxidative cleavage of polyisoprene chains

Rubber degrading bacteria must first cleave the water-insoluble high molecular weight polyisoprene molecules into low molecular weight products outside the cells. To this end, *S*. *cummioxidans* strain 35Y secretes two extracellular NR-degrading enzymes [16, 19], rubber oxygenases RoxA and RoxB. RoxB cleaves rubber into a mixture of C_20_ and higher oligoisoprenoids in an *endo*-type fashion thereby increasing the number of chain-ends. RoxA cleaves poly (*cis*-1, 4-isoprene) as well as the oligoisoprenoids derived from RoxB cleavage in an *exo-*type manner to the tri-isoprenoid ODTD. ODTD can be taken up by the bacterial cells directly or after oxidation of the terminal aldehyde group to a carboxylic acid group.

RoxA and RoxB harbour two haem-binding motifs (CXXCH) in the primary amino acid sequences [36] that enable a covalent attachment of the haem groups by the cytochrome *c* maturation proteins (see below) to the RoxA (RoxB) apo-peptides resulting in the mature holo-RoxA (holo-RoxB) enzymes (*c*-cytochromes). The presence of two *c*-type haem groups has already been confirmed by biophysical characterization of isolated RoxA protein [17] and by structure determination [18]. The published gene sequences of RoxA (GenBank accession number: KC980911.1) and RoxB (GenBank accession number: KY498024.1) enzymes were used to identify their locations in the draft genome of *S*. *cummioxidans* strain 35Y (*roxA*: STC_00358 (2,034 bp) and *roxB*: STC_02518 (2,043 bp)). The *roxA* and *roxB* genes had been identified and DNA-sequenced, previously [16, 36]. RoxA and RoxB exhibited high identities to the orthologous gene products of *R*. *gummiphilus*, i.e. RoxA (67%) and RoxB (LatA) (83%), respectively. However, the similarity of RoxA to RoxB was only moderate (30–40% identity) both for the *S*. *cummioxidans* strain 35Y and *R*. *gummiphilus* proteins. The biochemical properties of purified RoxA and RoxB of *R*. *gummiphilus* corresponded well with the homologous proteins of *S*. *cummioxidans* strain 35Y (data unpublished).

ODTD is the main end product of the RoxA/RoxB catalyzed extracellular cleavage of NR. The aldehyde group of ODTD must be oxidized to the corresponding acid extracellularly due to the high reactivity and toxicity of free aldehydes to living cells. The key enzyme for this step remains unknown [7, 8, 9] **(Fig 2)**. However, numerous putative oxidoreductases were present in the draft genome of *S*. *cummioxidans* strain 35Y, among which only three (STC_01691, STC_05669, STC_06528) **(S1 Dataset)** were predicted to be extracellular (SecP score ≥ 0.5, non-classically secreted without any signal peptide) and might be responsible for this function.

The incorporation of covalently attached haem groups during the maturation of *c-*type cytochromes, such as RoxA and RoxB or haem proteins of the respiratory chain is carried out by cytochrome *c* maturation (Ccm) proteins and DsbD [37]. We predicted a cluster of *ccm* genes, *ccmABC****_****EFGHI* with one hypothetical gene (STC_04494, between *ccmC* and *ccmE*) in *S*. *cummioxidans* strain 35Y **(Table C and Figure A in S1 Text)** using comparative genomics. STC_04494 showed high similarity (44% identity over 79% of its length) with putative haem exporter protein CcmD of *Methylococcus capsulatus* Bath (GenBank accession number: NC_002977.6). We therefore assume that STC_04494 represents the haem exporter protein (CcmD). In addition, a *dsbD* gene was also predicted in the draft genome sequence but was not part of the *ccm* gene cluster. DsbD proteins (thiol: disulfide interchange proteins) are involved in the assembly of functional *c*-type cytochromes in the periplasm of Gram-negative bacteria. Furthermore, we identified a putative promoter containing a transcriptional start site 36 bp upstream of the *ccm* gene cluster which presumably is responsible for the regulation of the expression of the Ccm proteins. Our data suggests that all nine Ccm proteins are encoded by one transcriptional unit (*ccmABCDEFGHI*).

### (2) Import of oligoisoprenes

Subsequent to the extracellular cleavage of polyisoprene via RoxA and RoxB and oxidation of ODTD to the corresponding fatty acid, the C_15_-tri-isoprenoid derivate has to be transported into the cells. The cell wall and cell membranes of Gram-positive and Gram-negative bacteria largely differ; moreover, the Lcp-derived primary degradation products of polyisoprene in Gram-positive rubber degrading bacteria are substantially larger (C_20_, C_25_ and higher oligoisoprenoids). Therefore, the transport proteins for the uptake of the primary rubber cleavage products might be different in Gram-positive and Gram-negative rubber degraders. In agreement with this assumption, we were unable to detect the genes for a complete transmembrane substrate uptake protein system of the YrbE/Mce (mammalian cell entry) type. In contrast, the genomes of the Gram-positive rubber degrading species *G*. *polyisoprenivorans* VH2 [7] and *N*. *nova* SH22a [8, 9] have genes for multiple YrbE*/*Mce clusters. One or several of these are thought to be specific for the uptake of rubber degradation products **(Table 3)**. Thus, *S*. *cummioxidans* strain 35Y might have evolved a different transport mechanism.

**Table 3:** Distribution and loci of mammalian cell entry cluster related putative genes in the draft genome of *Steroidobacter cummioxidans* strain 35Y.

Gene/Gene product	Locus
***Mce* (Mammalian cell entry protein)**	STC_00777, STC_01556, STC_05337
***YrbE* (ABC transporter permease)**	STC_00779, STC_01557, STC_05339

**Fig 3:**
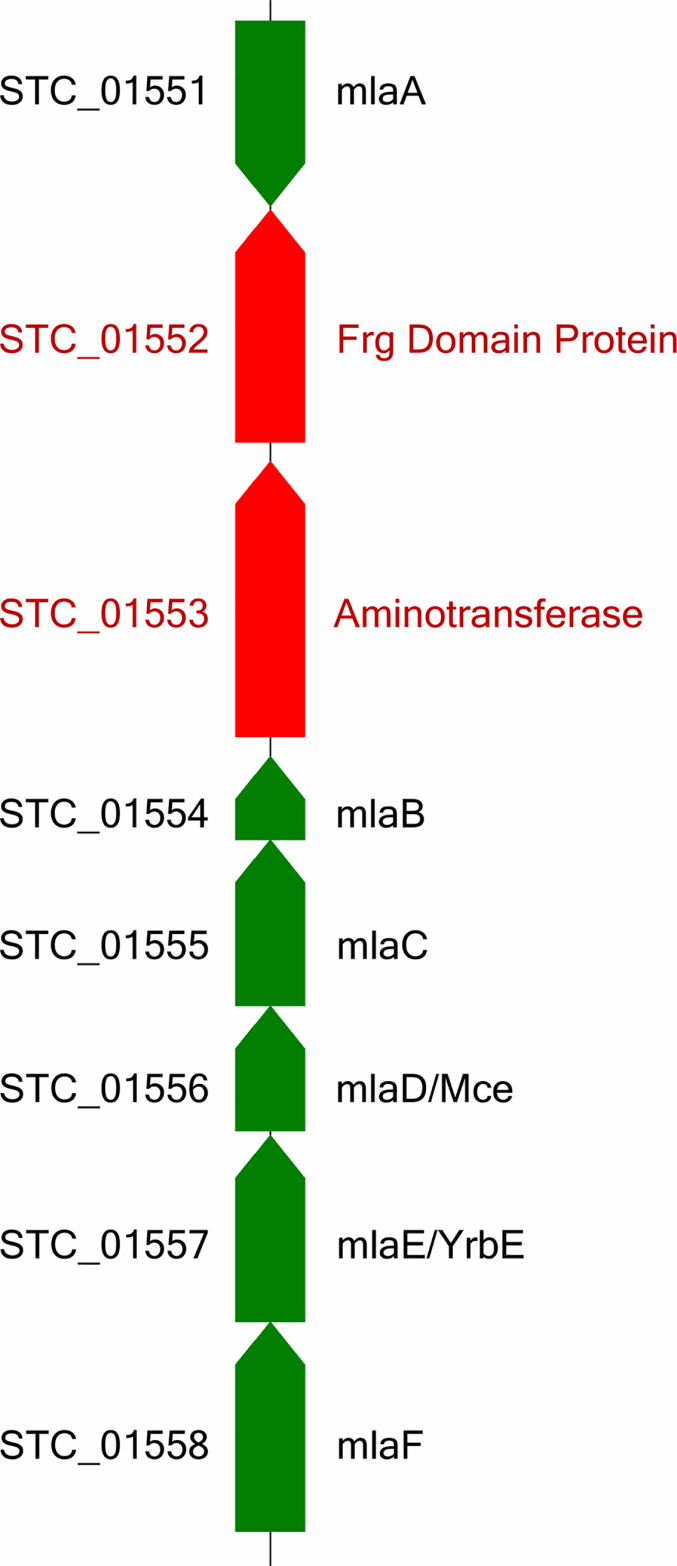
Putative gene cluster of maintenance of outer membrane lipid asymmetry pathway in the draft genome of *Steroidobacter cummioxidans* strain 35Y. STC_01552 and STC_01553 are intervening genes in this *Mla* gene cluster (shown in red). Mla: An ABC transport system that maintains lipid asymmetry. For functional description of *mla* genes, refer to **Table D in S1 Text**.

A novel ABC transport system for intermembrane phospholipid trafficking, known as the maintenance of bacterial outer membrane lipid asymmetry (Mla) pathway, which maintains lipid asymmetry and is encoded by six genes, (*mlaA*, *mlaB*, *mlaC*, *mlaD*, *mlaE*, and *mlaF)* has been described for Gram-negative bacteria [38]. The entire Mla pathway was predicted to be conserved in *S*. *rubberoxidans* strain 35Y **(Table D in S1 Text and Fig 3)** using comparative genomics. In addition, Gram-negative bacteria also possess two families of substrate-specific outer membrane transporters involved in fatty-acid uptake, namely TonB-dependent outer membrane receptors (TBDRs) and long-chain fatty acid transporters (FadL family) [39]. For the transport of exogenous fatty acids, *fadD* encoding fatty acyl coenzyme A (CoA) synthetase (FACS) is found in Gram-negative bacteria [40]. It is also suggested that FadL and FACS function together as a fatty acid transport apparatus in Gram-negative bacteria. *S*. *cummioxidans* strain 35Y harboured one copy of a putative *fadL* family transporter gene and eleven copies of putative *FACS* genes **(Table E in S1 Text)**. In addition, numerous copies of putative TBDR genes were also present in the draft genome. The availability of the draft genome sequence of *S*. *cummioxidans* strain 35Y now enables the performance of proteome studies to find experimental evidence if and which of the identified genes are specifically upregulated in cells grown on NR.

### (3) β-oxidation

β-oxidation is an iterative biological process which is responsible for the breakdown of fatty acids as a part of lipid metabolism. Recently, evidence for the involvement of β-oxidation enzymes in the catabolism of rubber was discussed for the Gram-positive rubber degrading species *Streptomyces coelicolor* 1A [41], *G*. *polyisoprenivorans* VH2 [7], and *N*. *nova* SH22a [8, 9]. The entire set of genes involved in the β-oxidation pathway was predicted to be conserved in *S*. *cummioxidans* strain 35Y **(Table F in S1 Text and Fig 2)** using comparative genomics.

### (4) Acetyl-CoA and propionyl-CoA metabolism

The cleavage products of NR-degradation have to be channelled into the tricarboxylic acid (TCA) cycle in order to fuel the respiratory chain. The proposed NR-degradation pathway is similar to polyunsaturated fatty acid degradation and released two products, acetyl-CoA and propionyl-CoA [7, 9, 41] **(Fig 2)**. Acetyl-CoA enters directly into the TCA cycle and/or the glyoxylate bypass, whereas propionyl-CoA is channelled indirectly via the methylcitrate pathway and the methylmalonyl-CoA pathway by a cascade of biochemical reactions to convert it into succinic acid. Towards this, the entire set of genes, that could fulfil these functions, was identified in the draft genome of *S*. *cummioxidans* strain 35Y **(Tables G and H in S1 Text and Fig 2**) using comparative genomics.

### (5) Anaplerotic reactions and gluconeogenesis

In the absence or limited availability of sugars as the carbon and energy source, microorganisms employ conversion of intermediates of TCA cycle or the glyoxylate bypass to phosphoenolpyruvate (PEP). Based on genome analysis, entire sets of genes for TCA cycle and glyoxylate bypass were predicted in the draft genome of *S*. *cummioxidans* strain 35Y **(Table I in S1 Text)**. In addition to this, orthologs of enzymes, including malic enzyme and PEP synthase, which catalyze the alternative routes to PEP biosynthesis [42, 43] were also predicted in the draft genome of *S*. *cummioxidans* strain 35Y **(Fig 2)**. PEP acts as a substrate for several enzymatic reactions participating in significant cellular processes, including gluconeogenesis, glyceroneogenesis, amino acid synthesis, and anaplerosis of the TCA or glyoxylate cycle [44]. Phosphoenolpyruvate carboxykinase (PEPCK) is the key enzyme of this step **(Fig 2)** and catalyzes the guanosine or adenosine mononucleotide-dependent reversible conversion of PEP and oxaloacetic acid. In many bacteria, this enzyme is essential for their growth or survival primarily when grown on organic acids as sole carbon and energy source. Towards this, *S*. *cummioxidans* strain 35Y harbours one gene coding for a putative PEP carboxykinase **(Table J in S1 Text)**.

### Biosynthesis of industrially relevant secondary metabolites

The class *Gammaproteobacteria* includes several industrially relevant species that produce a wide range of economically-important metabolites, including PHAs, xanthan, xanthomonadin etc. PHA granules are composed of a polymer core which is surrounded by a complex proteinaceous surface layer consisting of at least five types of PHA granule-associated proteins (PGAPs) [45]. *S*. *cummioxidans* strain 35Y was predicted to harbour the structural genes, including type III PHA synthase (PhaC-PhaE) and a transcriptional regulator (PhaR), for the biosynthesis of short-chain length PHAs **(Table K in S1 Text)** using comparative genomics. However, genes for other PHA granule associated proteins PGAPs, such as phasins (PhaPs) [46, 47] and PHB depolymerases [PhaZ(a/d)s] [48, 49], involved in PHA accumulation and homeostasis, were not found. This pointed to the absence of a complete PHA granules assembly pathway and was in agreement with the inability to detect PHA granules upon cultivation of the cells under otherwise PHA-permissive conditions. **(Figure Ba in S1 Text)**. Also, a complete set of genes for the biosynthesis of other carbon storage materials, including granulose **(Table L in S1 Text)** and cellulose **(Table M in S1 Text)** were predicted. In addition, a few genes of the xanthan gum biosynthesis pathway were predicted in the draft genome of *S*. *cummioxidans* strain 35Y **(Table N in S1 Text)**.

Based on genome analysis, the draft genome of *S*. *cummioxidans* strain 35Y was predicted to harbour polyphosphate kinase (*ppk*) and polyphosphatase (*ppx*) genes involved in the biosynthesis of polyP containing granules **(Table O in S1 Text)**. The ability to synthesize polyP was confirmed by staining of the cells with DAPI and the appearance of bright fluorescent granules in the cells **(Figure Bb in S1 Text).**

All genes necessary for the complete biosynthetic pathway of xanthomonadin **(Figure C and Table P in S1 Text)** were predicted in the draft genome of *S*. *cummioxidans* strain 35Y using comparative genomics. Xanthomonadin is a characteristic yellow pigment produced by the members of the genus *Xanthomonas*. The presence of xanthomonadin had been previously confirmed by solvent-extraction of a yellow compound that revealed an absorption maximum at 441 nm similar to that of xanthomonadin of authentic *Xanthomonas* species [50]. An *m/z* value of 560 and a molecular formula of C_27_H_28_Br_2_O_3_ was determined by mass spectrometry of the thin layer chromatography (TLC) purified pigment. These properties corresponded well with the xanthomonadin pigment group-7 [51] and explain the typical yellow colour of *S*. *cummioxidans* strain 35Y colonies on solid media.

## Conclusions

This study characterized strain 35Y based on genotypic, physiological, and biochemical features and supported its description as a novel species of the genus *Steroidobacter*, for which the name *Steroidobacter cummioxidans* sp. nov., strain 35Y^T^ is proposed. However, it has not escaped our notice that some of the characteristic physiological, biochemical, and genomic features of strain 35Y presented in this study can be used as major differentiating features for not only species demarcation but also for the genus demarcation of strain 35Y from the genus *Steroidobacter*. However, this requires further support from comprehensive taxonomic analysis using additional experimental and *in silico* data. Further, a comprehensive *in silico* exploration of the draft genome of *S*. *cummioxidans* strain 35Y has led to the elucidation of the putative pathway for the break-down of NR by a Gram-negative rubber degrading species which differs from that of Gram-positive bacteria primarily in the nature of the uptake system for the primary NR-degradation products. The uptake system of *S*. *cummioxidans* strain 35Y has to operate with a product of a defined shorter length (acid of tri-isoprenoid cleavage product ODTD) in comparison to the acids of tetra- and higher-oligoisoprenoids of Gram-positive rubber degraders. In addition, *S*. *cummioxidans* strain 35Y is also predicted to possess a biosynthetic potential to synthesize secondary metabolites which can have wide industrial applications. In brief, the present study is significant in terms of characterizing a novel species of a Gram-negative bacteria and elucidating its NR-degradation pathway which will aid future studies to exploit the biodegradation of rubber.

## Supporting information

S1 Dataset Genome features of *Steroidobacter cummioxidans* strain 35Y. (XLS)

S1 Text Supporting methods, results, references, figures, and tables of the current study. (DOCX)
