# Correction: Metabolic and taxonomic insights into the Gram-negative natural rubber degrading bacterium *Steroidobacter cummioxidans* sp. nov., strain 35Y

**DOI:** 10.1371/journal.pone.0200399

**Published:** 2018-07-09

**Authors:** Vikas Sharma, Gabriele Siedenburg, Jakob Birke, Fauzul Mobeen, Dieter Jendrossek, Tulika Prakash

There are errors in the Funding section. The correct funding information is as follows: This work was supported by a grant of the Deutsche Forschungsgemeinschaft to DJ (DFG Je 152/17-1) and Vikas Sharma is a recipient of a research fellowship from the Ministry of Human Resource Development (MHRD), India (http://mhrd.gov.in/). The funders had no role in study design, data collection and analysis, decision to publish, or preparation of the manuscript.
