# Supplementary material for: Metabolic and taxonomic insights into the Gram-negative natural rubber degrading bacterium Steroidobacter cummioxidans sp. nov., strain 35Y
Source: PLoS One. 2018 May 31;13(5):e0197448. doi: 10.1371/journal.pone.0197448 (PMC5979037; doi:10.1371/journal.pone.0197448)
Supplement: S1 Text — (DOCX) [file pone.0197448.s002.docx]

**Metabolic and taxonomic insights into the Gram-negative natural rubber degrading bacterium *Steroidobacter cummioxidans* sp. nov., strain** **35Y**

**Vikas Sharma^1^, Gabriele Siedenburg^2,#a^, Jakob Birke^2^, Fauzul Mobeen^1^, Dieter Jendrossek^2^, Tulika Prakash^1*^**

^1^School of Basic Sciences, Indian Institute of Technology (IIT) Mandi, Mandi, India

^2^Institute of Microbiology, University of Stuttgart, Stuttgart, Germany

^#a^Current Address: University of Duisburg-Essen, Essen, Germany

^*^Corresponding author

Email: tulika@iitmandi.ac.in (TP)

**S1 Text. Supporting methods, results, references, figures, and tables of the current study.**

# **Methods**

**Metabolic pathway analysis of *Steroidobacter* *denitrificans* DSM 18526**

The publically available metabolic pathways of *S. denitrificans* DSM 18526 were obtained from Kyoto Encyclopedia of Genes and Genomes (KEGG) database for their comparison with the metabolic pathways of *Steroidobacter cummioxidans* strain 35Y.

**Results and discussion**

**Basic genome features**

The draft genome of *S. cummioxidans* strain 35Y was 7,936,208 bp in length and was assembled into 126 contigs with DNA G+C content of 60.94 mol%. The draft genome contained 6,906 predicted protein coding genes, 7 rRNA genes, and 47 tRNA genes **(S1 Dataset).** Of the 6,906 proteins, tentative functions were predicted for 5,475 proteins using similarity based approaches **(S1 Dataset)**. The remaining 1,431 proteins did not exhibit significant similarity to proteins of any known function and thus remained as hypothetical or uncharacterized **(S1 Dataset)**. For further functional analysis, the predicted proteins were classified into different functional classes using Cluster of Orthologous Groups (COGs). Out of 6,906 proteins, 2,637 were mapped to 1,570 unique COGs which were further classified into metabolism (35.61%), cellular processes and signaling (17.29%), information storage and processing (15.24%), and poorly characterized (18.54%) functional classes **(Figure D in S1 Text)**. The remaining 13.31% proteins were assigned to more than one COGs categories and were grouped into “multiple classes” **(Figure D in S1 Text)**. In order to explore the pathway mapping of the draft genome, the predicted proteins were annotated using KEGG database. A total of 2,892 proteins (41.87%) showed similarity with 1,918 unique KEGG identifiers corresponding to different metabolic pathways. In the draft genome of *S. cummioxidans* strain 35Y, 479 proteins were predicted as putative membrane transporters. Also, 909 proteins were predicted as putative virulence factors in the draft genome of *S. cummioxidans* strain 35Y. In addition, 6,906 proteins were classified into five categories based upon subcellular localization, including cytoplasmic (37.85%), cytoplasmic membrane (20.50%), extracellular (1.51%), outer membrane (3.82%), and periplasmic (2.43%) whereas, 33.88% remained unclassified.

**Additional genome and metabolic features**

Based on genome analysis, the draft genome of *S. cummioxidans* strain 35Y was predicted to harbour a cluster of genes for urea transporters located adjacent to the urease operon **(Tables Q and R in S1 Text)**. Urea transporters are found in very few bacteria, such as *Helicobacte*r species and cyanobacteria [1]. These bacteria use this system of urea metabolism for gradual adaptations in a given environment, including gut and nitrogen-limiting conditions. This implied that *S. cummioxidans* strain 35Y might be able to metabolize urea under nitrogen limiting conditions in stressful environment. In concordance with this, *S. cummioxidans* strain 35Y was able to use urea as a sole source of nitrogen (data unpublished). In addition, *S. cummioxidans* strain 35Y was predicted to possess candidate genes encoding an osmoprotectant transport system comprising *opuC*/*opuBD* (STC_1279) and *opuA* (STC_1280) genes. This system plays a prominent role in osmoregulatory functions in a few bacteria, including *Pseudomonas syringae* [2] and *Bacillus subtilis* [3]*.* This suggested that *S. cummioxidans* strain 35Y might also use this osmoprotectant transport system to cope with osmotic stress conditions. In addition, *S. cummioxidans* strain 35Y also harboured four putative gene copies for polyketide synthase which is an essential enzyme for the biosynthesis of numerous secondary metabolites, including antibiotics and antifungals **(Table S in S1 Text)**.

Based on genome analysis, the draft genome of *S. cummioxidans* strain 35Y was predicted to contain several genes encoding enzymes involved in the transport and metabolism of carbohydrates **(Table T in S1 Text and S1 Dataset)** which are exclusively associated with terrestrial plant hemicellulose [4]. The presence of glycoside hydrolase (GH) in the draft genome of *S. cummioxidans* strain 35Y supported environmental-adaptation of *S. cummioxidans* strain 35Y as this enzyme class has been implicated in the degradation of plant organic matter thus depicting its important soil functions, including decomposing the plant-residues and recycling the soil nutrients [5].

Using comparative genomics, *S. cummioxidans* strain 35Y was predicted to have a cluster of 14 genes involved in biosynthesis of xanthomonadin **(Figure C and Table P in S1 Text)** whereas *S. denitrificans* DSM 18526 was predicted to possess only 3 genes (3-ketoacyl-AP reductase (AMN48017.1), 3-oxoacyl-ACP synthase (AMN47588.1, AMN46991.1), and pteridine-dependent deoxygenase (AMN45628.1)) of xanthomonadin cluster.

**Microscopy analysis**

The cellular dimensions of *S. cummioxidans* strain 35Y were 0.4 to 0.6 μm in width and 2 to 5 µm in length but cells occasionally could become considerably longer (up to 10 μm) **(Figure Bc in S1 Text)**.

**Central energy metabolism**

*S. cummioxidans* strain 35Y has a strictly aerobic metabolism [6] and its draft genome carried putative genes for aerobic respiration, including genes for Embden-Meyerhof pathway for glycolysis, pyruvate decarboxylation, tricarboxylic acid cycle, and oxidative phosphorylation based on genome analysis. Along with catabolic glycolysis, *S. cummioxidans* strain 35Y was also predicted to harbour genes for pentose phosphate pathway, an anabolic process which produces biologically significant molecules, including nicotinamide adenine dinucleotide phosphate (NADPH), ribose 5-phosphate, and erythrose 4-phosphate which are required for the biosynthesis of fatty acid, nucleotides, and aromatic amino acids, respectively **(S1 Dataset)**. In addition to glucose-metabolism, it also carried putative genes for the metabolism of other sugars, including fructose, sucrose, mannose, galactose, and starch. Besides the central carbohydrate metabolism, the draft genome of *S. cummioxidans* strain 35Y was also predicted to contain genes for the synthesis of all 20 proteinogenic amino acids, all nucleotides, fatty acid metabolism, and different cofactors and vitamins required in various catalytic biological processes **(S1 Dataset)**.

**Prophages and insertion elements**

PHAge Search Tool (PHAST) analysis predicted two regions comprising incomplete prophages in the draft genome of *S. cummioxidans* strain 35Y. One region was 10.1 kb in length and comprised 8 genes whereas another region was 9.4 kb in length and comprised 9 genes **(S1 Dataset)**. The functional analysis of these prophages predicted Gram-negative bacteria as their host and lytic cycle as a primary mode of reproduction. Further, Isfinder predicted 90 insertion elements (IEs), among which ISBma3 (Family IS110), ISXci1 (Family IS21), ISRso19 (Family IS21), TnXo19 (Family Tn3), ISPa25 (Family IS110) were the most abundant IEs **(S1 Dataset)**. The presence of prophages and insertion elements in the draft genome of *S. cummioxidans* strain 35Y depicted the transformation and recombination events in its genome which are integrated part of evolutionary process in terms of gain or loss of certain genes. This might have also contributed to its genome size which is largest within the genus *Steroidobacter*. In addition, CRISPRFinder predicted at least 3 CRISPR loci in the draft genome of *S. cummioxidans* strain 35Y which depicted its genome readability for bacteriophage exposure and genome-editing **(S1 Dataset)**.

**Antibiotic resistant genes**

To predict the antibiotic resistant genes in the draft genome of *S. cummioxidans* strain 35Y, mining for the antibiotic resistant homologs was carried out in the CARD database. No antibiotic resistant gene was predicted on perfect criteria. However, at strict criteria, it predicted 50 genes with functions in antibiotic resistance among which *adeG* (inner membrane transporter of the AdeFGH multidrug efflux complex) and *mdtC* (multidrug transporter) were the most abundant functions **(S1 Dataset)**.

**Domain analysis of RoxA and RoxB**

Both RoxA and RoxB enzymes of *S. cummioxidans* strain 35Y were predicted to possess the cytochrome domains **(S1 Dataset)** using *in silico* analysis.

**Comparison of metabolic pathways of *S. denitrificans* DSM 18526 and *S. cummioxidans* strain** **35Y**

Upon extensive mining of the publically available metabolic pathways (KEGG) of only available genome of the genus *Steroidobacter* (*S. denitrificans* DSM 18526), we could not detect the metabolism related genes corresponding to several amino acids, including cysteine, serine, arginine, methionine, and histidine. In contrast, the draft genome of *S. cummioxidans* strain 35Y was predicted to contain the genes for the metabolism of all these amino acids **(S1 Dataset)** thus could utilize a wide range of carbon sources, such as sugars, sugar acids, acids, and hydrocarbons and grew well on amino acids and complex media [7].

# **References**

1. Esteva-Font C, Anderson MO, Verkman AS. Urea transporter proteins as targets for small-molecule diuretics. Nat Rev Nephrol. 2015;11:113-123.
2. Chen C, Beattie GA. Characterization of the osmoprotectant transporter OpuC from *Pseudomonas syringae* and demonstration that cystathionine-β-synthase domains are required for its osmoregulatory function. J Bacteriol. 2007;189:6901-6912.
3. Pittelkow M, Tschapek B, Smits SH, Schmitt L, Bremer E. The crystal structure of the substrate-binding protein OpuBC from *Bacillus subtilis* in complex with choline. J Mol Biol. 2011;411:53-67.
4. Kolton M, Sela N, Elad Y, Cytryn E. Comparative genomic analysis indicates that niche adaptation of terrestrial Flavobacteria is strongly linked to plant glycan metabolism. PLoS One. 2013;8:e76704.
5. Allgaier M, Reddy A, Park JI, Ivanova N, D'haeseleer P, Lowry S, et al. Targeted discovery of glycoside hydrolases from a switchgrass-adapted compost community. PLoS One. 2010;5:e8812.
6. Tsuchii A, Takeda K. Rubber-degrading enzyme from a bacterial culture. Appl Environ Microbiol. 1990;56:269-274.
7. Kerkhoff K. Ph.D. thesis. Molekularbiologische und biochemische Untersuchungen zum bakteriellen Naturkautschuk-Abbau, sowie Charakterisierung eines dazu behähigten Bakteriums: Niedersächsische Staats-und Universitätsbibliothek; 2000.


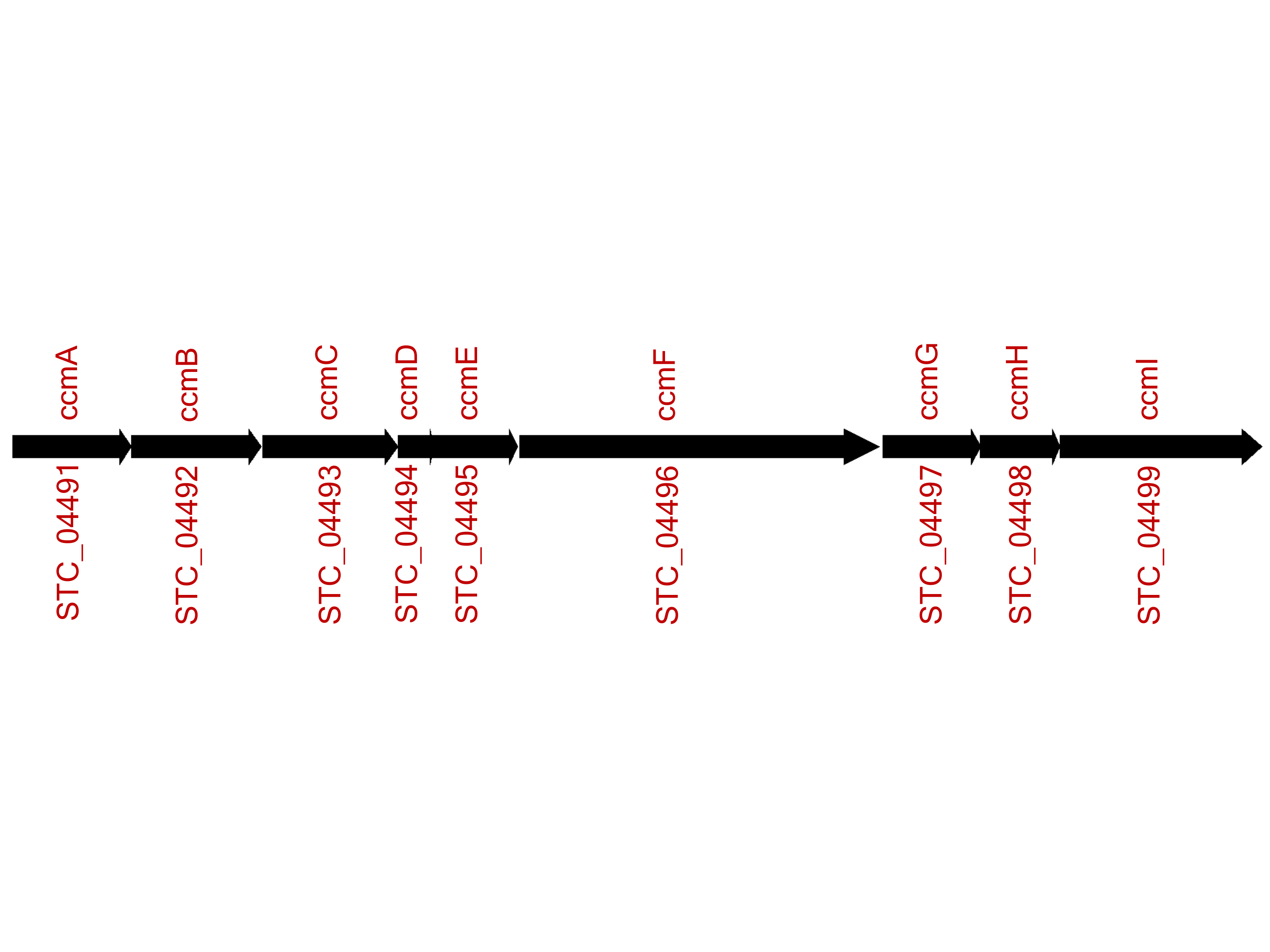


**Figure A. Putative cytochrome *c* maturation cluster located in the draft genome of *Steroidobacter cummioxidans* strain 35Y.** *ccmA*: ATP-binding haem export protein; *ccmB*: Haem exporter protein; *ccmC*: Haem exporter protein; *ccmD*: Haem exporter protein; *ccmE*: Haem chaperone; *ccmF*: holo*CcmE* binding protein; *ccmG*: reduction and transfer of apocyt *c* to the haem b ligation site; *ccmH*: apocyt *c* binding protein; *ccmI*: apocyt *c* binding protein.


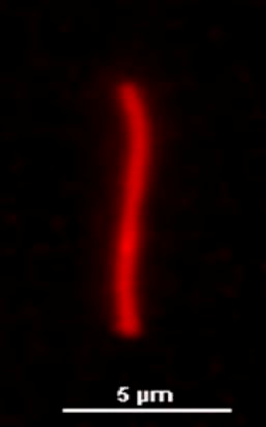

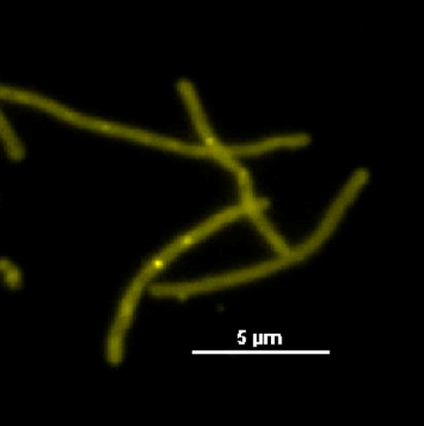

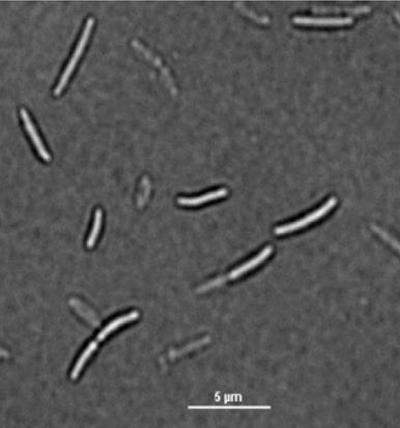


**(c)**

**(b)**

**(a)**

**Figure B. Micrographs of cells of *Steroidobacter cummioxidans* strain** **35Y** **(a)** stained with Nile red showing no polyhydroxyalkanoate granules using fluorescent microscopy, **(b)** stained with 4’,6-diamino-2-phenylindole 2HCl, showing bright spots of polyphosphate granules using fluorescent microscopy, and **(c)** using bright field microscopy.

**
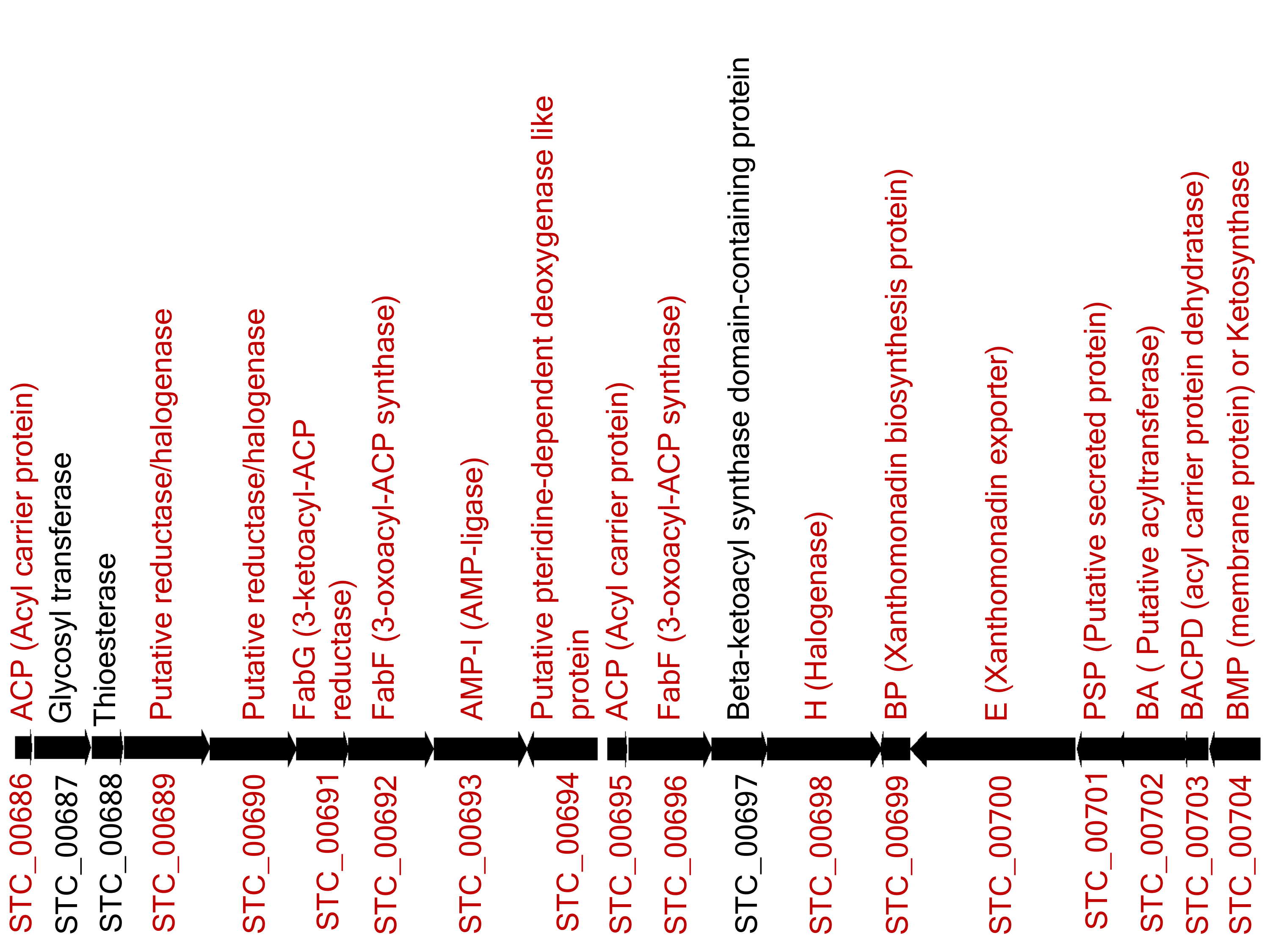
**

**Figure C. Putative xanthomonadin gene cluster located in the draft genome of *Steroidobacter cummioxidans* strain** **35Y.** STC_00687, STC_00688, STC_00697 (black fonts) are intervening genes in this xanthomonadin gene cluster.


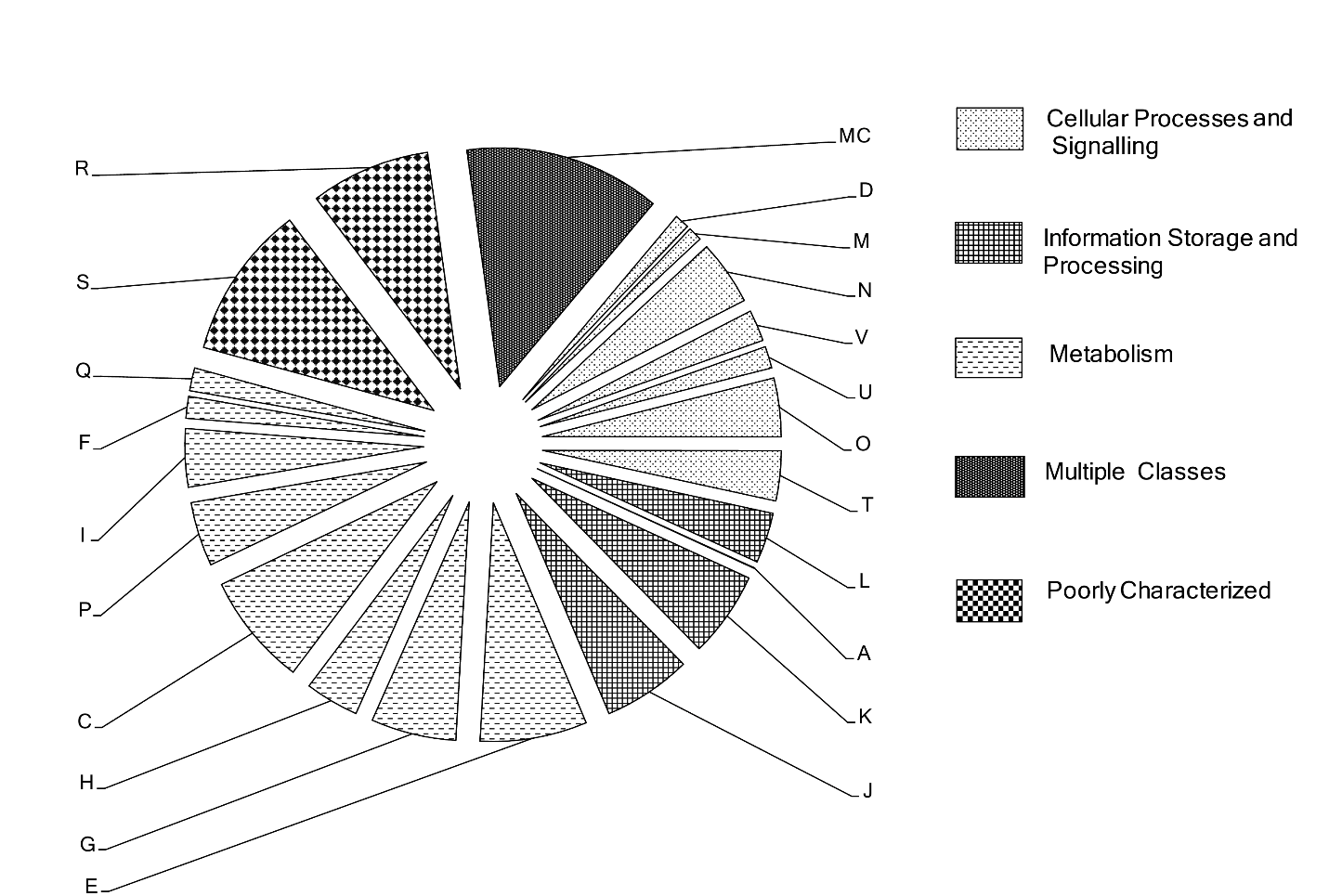


**Figure D. Distribution of functional Cluster of Orthologous Groups classes in the draft genome of *Steroidobacter cummioxidans* strain 35Y.** D:Cell cycle control, cell division, chromosome partitioning; M:Cell motility; N:Cell wall/membrane/envelope biogenesis; V:Defense mechanisms; U:Intracellular trafficking, secretion, and vesicular transport; O:Posttranslational modification, protein turnover, chaperones; T:Signal transduction mechanisms; L:Replication, recombination and repair; A:RNA processing and modification; K:Transcription; J:Translation, ribosomal structure and biogenesis; E:Amino acid transport and metabolism; G:Carbohydrate transport and metabolism; H:Coenzyme transport and metabolism; C:Energy production and conversion; P:Inorganic ion transport and metabolism; I:Lipid transport and metabolism; F:Nucleotide transport and metabolism; Q:Secondary metabolites biosynthesis, transport and catabolism; S:Function unknown; R:General function prediction only; MC: Multiple classes.

**Table A. List of bacteria used in this study for the comparative genomics and prediction of orthologs in the draft genome of *Steroidobacter cummioxidans* strain** **35Y.**

| **Organism** | **Accession Number** |
| --- | --- |
| *Acidithiobacillus ferrivorans* SS3 | CP002985.1 |
| *Actinobacillus suis* H91-0380 | CP003875.1 |
| *Algiphilus aromaticivorans* DG1253 | NZ_JPOG00000000.1 |
| *Azoarcus* sp. BH72 | NC_008702.1 |
| *Chitinophaga pinensis* DSM 2588 | NC_013132.1 |
| *Dechloromonas aromatic* RCB | CP000089.1 |
| *Desulfobacterium autotrophicum* HRM2 | NC_012108.1 |
| *Escherichia coli* O25b:H4 | CP015085.1 |
| *Flavobacterium johnsoniae* UW101 | NZ_MUGZ00000000.1 |
| *Gordonia bronchialis* DSM 43247 | NC_013441.1 |
| *Gordonia* sp. KTR9 | NC_018581.1 |
| *Gordonia polyisoprenivorans* VH2 | NC_016906.1 |
| *Haliangium ochraceum* DSM 14365 | NC_013440.1 |
| *Hydrocarboniphaga effusa* AP103 | NZ_AKGD00000000.1 |
| *Methylococcus capsulatus* Bath | NC_002977.6 |
| *Myxococcus fulvus* HW-1 | CP002830.1 |
| *Nevskia ramosa* DSM 11499 | NZ_ATVI00000000.1 |
| *Nevskia soli* DSM 19509 | KL543998.1 |
| *Nocardia nova* SH22a | NZ_CP006850.1 |
| *Polycyclovorans algicola* TG408 | NZ_JOMH00000000.1 |
| *Pseudomonas* sp. NBRC 111125 | NZ_BCBB01000150.1 |
| *Rhodobacter sphaeroides* 2.4.1 | NC_007493.2 |
| *Sideroxydans lithotrophicus* ES-1 | NC_013959.1 |
| *Singularimonas variicoloris* DSM 15731 | NZ_ARNM00000000.1 |
| *Solimonas flava* DSM 18980 | NZ_AUFV00000000.1 |
| *Solimonas soli* DSM 21787 | NZ_AXDW00000000.1 |
| *Stenotrophomonas maltophilia* JV3 | CP002986.1 |
| *Stenotrophomonas maltophilia* K279a | NC_010943.1 |
| *Stenotrophomonas maltophilia* R551-3 | NC_011071.1 |
| *Steroidobacter denitrificans* DSM 18526 | NZ_CP011971.1 |
| *Variovorax paradoxus* EPS | NC_014931.1 |
| *Variovorax paradoxus* S110 | NC_012791.1 and NC_012792.1 |
| *Xanthomonas albilineans* GPE PC73 | NC_013722.1 |
| *Xanthomonas arboricola* pv*. pruni* MAFF 311562 | BAVB00000000.1 |
| *Xanthomonas axonopodis* pv*. citri* 306 | NC_003919.1 |
| *Xanthomonas axonopodis* pv*. citrumelo* F1 | NC_016010.1 |
| *Xanthomonas axonopodis* Xac29-1 | NC_020800.1 |
| *Xanthomonas campestris* pv*. campestris* 8004 | NC_007086.1 |
| *Xanthomonas campestris* pv*.* *campestris* ATCC 33913 | JX453143.1 |
| *Xanthomonas campestris* pv*. campestris* B100 | NC_010688.1 |
| *Xanthomonas campestris* pv*. raphani* 756C | CP002789.1 |
| *Xanthomonas campestris* pv*.* *vesicatoria* 85-10 | NZ_CP017190.1 |
| *Xanthomonas citri* subsp*.* *citri* Aw12879 | CP003778.1 |
| *Xanthomonas fuscans* subsp. *Fuscans* XCP631 | NZ_JXLW00000000.2 |
| *Xanthomonas oryzae* pv*. oryzae* KACC 10331 | AE013598.1 |
| *Xanthomonas oryzae* pv*. oryzae* MAFF 311018 | NC_007705.1 |
| *Xanthomonas oryzae* pv*. oryzae* PXO99A | NC_010717.2 |
| *Xanthomonas oryzae* pv*. oryzicola* BLS256 | NC_017267.2 |

**Table B. 16S rRNA gene sequence identities of *Steroidobacter cummioxidans* strain 35Y with members of the family *Sinobacteraceae*.**

| **Organism** | **Identity (%)** |
| --- | --- |
| *Steroidobacter flavus* CPCC 100154 | 98.27 |
| *Steroidobacter agariperforans* KA5-B | 98.19 |
| *Steroidobacter denitrificans* DSM 18526 | 97.78 |
| *Povalibacter uvarum* Zumi 37 | 97.66 |
| *Steroidobacter denitrificans* FS | 97.59 |
| *Povalibacter* sp. MS35 | 95.19 |
| *Steroidobacter* sp. WWH78 | 93.5 |
| *Steroidobacter* sp. JC2953 | 89.68 |
| *Solimonas flava* DSM 18980 | 89.22 |
| *Steroidobacter* sp. JC2986 | 89.05 |
| *Fontimonas* sp. ZC84 | 88.74 |
| *Polycyclovorans* sp. ZY33 | 88.51 |
| *Solimonas aquatica* DSM 25927 | 88.48 |
| *Panacagrimonas perspica* Gsoil 142 | 88.41 |
| *Sinimarinibacterium flocculans* Za3-11 | 88.25 |
| *Alkanibacter difficilis* MN154.3 | 88.2 |
| *Solimonas variicoloris* DSM 15731 | 88.15 |
| *Sinimarinibacterium flocculans* NH6-24 | 88.12 |
| *Solimonas aquatica* NAA16 | 88.11 |
| *Hydrocarboniphaga effusa* AP103 | 88.05 |
| *Nevskia* sp. Seoho-38 | 88.05 |
| *Hydrocarboniphaga* sp. FSBRY8 | 87.96 |
| *Hydrocarboniphaga* sp. FSBRY7 | 87.95 |
| *Solimonas variicoloris* MN28 | 87.93 |
| *Hydrocarboniphaga daqingensis* B2-9 | 87.91 |
| *Solimonas flava* CW-KD 4 | 87.87 |
| *Solimonas soli* DSM 21787 | 87.83 |
| *Hydrocarboniphaga daqingensis* C7 | 87.77 |
| *Nevskia* sp. KNF004 | 87.74 |
| *Fontimonas thermophila* HA-01 | 87.71 |
| *Hydrocarboniphaga* sp. 2Atol2 | 87.69 |
| *Solimonas* sp. p11(2011) | 87.67 |
| *Hydrocarboniphaga* sp. LeSa36d | 87.67 |
| *Hydrocarboniphaga daqingensis* NBRC 104238 | 87.64 |
| *Solimonas soli* DCY12 | 87.62 |
| *Nevskia soli* DSM 19509 | 87.61 |
| *Nevskia terrae* KIS13-15 | 87.55 |
| *Nevskia* sp. Iso-59 | 87.52 |
| *Hydrocarboniphaga* sp. BUE | 87.34 |
| *Nevskia soli* GR15-1 | 87.29 |
| *Nevskia aquatilis* F2-178 | 87.27 |
| *Nevskia persephonica* G6-54 | 87.25 |
| *Nevskia persephonica* G6M-30 | 87.25 |
| *Nevskia soli* NBRC 106401 | 87.19 |
| *Nevskia ramosa* DSM 11499 | 87.18 |
| *Nevskia* sp. KNF011 | 87.11 |
| *Solimonas* sp. CDMK | 87.11 |
| *Nevskia* sp. KNF010 | 87.03 |
| *Nevskia* sp. KNF003 | 87.03 |
| *Nevskia* sp. KNF014 | 87.01 |
| *Nevskia aquatilis F2-63* | 86.98 |
| *Solimonas* sp. HR-BB | 86.96 |
| *Solimonas terrae* KIS83-12 | 86.9 |
| *Nevskia* sp. KNF013 | 86.8 |
| *Nevskia* sp. KNF012 | 86.75 |
| *Polycyclovorans algicola* TG408 | 86.64 |

**Table C. Distribution and loci of cytochrome *c* maturation related putative genes in the draft genome of *Steroidobacter cummioxidans* strain** **35Y.**

| **Gene/Gene product** | **Locus** |
| --- | --- |
| ccmA | STC_04491 |
| ccmB | STC_04492 |
| ccmC | STC_04493 |
| ccmD | STC_04494* |
| ccmE | STC_04495 |
| ccmF | STC_04496 |
| ccmG | STC_04497 |
| ccmH | STC_04498 |
| ccmI | STC_04499* |
| DsbD | STC_05674 |

* implies the orthologous genes identified by comparative approach used in this study.

**Table D. Distribution and loci of maintenance of outer membrane lipid asymmetry pathway related putative genes in the draft genome of *Steroidobacter cummioxidans* strain** **35Y.**

| **Gene/Gene product** | **Locus** |
| --- | --- |
| *mlaA* (Phospholipid-binding lipoprotein) | STC_01551 |
| *mlaB* (Phospholipid transport system transporter-binding protein) | STC_01554 |
| mlaC (Phospholipid transport system substrate-binding protein) | STC_01555, STC_05482 |
| mlaD (Phospholipid/cholesterol/gamma-HCH transport system substrate-binding protein) | STC_00777, STC_01556,  STC_05337 |
| mlaE (Phospholipid/cholesterol/gamma-HCH transport system permease protein) | STC_00779, STC_01557,  STC_05339 |
| mlaF (Phospholipid/cholesterol/gamma-HCH transport system ATP-binding protein) | STC_00778, STC_01558,  STC_05338 |

**Table E. Distribution and loci of fatty acyl coenzyme A synthetase and long-chain fatty acid transporters FadL related putative genes in the draft genome of *Steroidobacter cummioxidans* strain 35Y.**

| **Gene/Gene product** | **Locus** |
| --- | --- |
| FadL (Long-chain fatty acid transport protein) | STC_01277 |
| FACS (Acyl coenzyme A (CoA) synthetase (FACS; fatty acid CoA ligase [AMP forming])) | STC_00701, STC_01699, STC_03638,  STC_04107, STC_04684, STC_04751,  STC_04777, STC_05511, STC_05561,  STC_06573, STC_06899 |

**Table F. Distribution and loci of β-oxidation related putative genes in the draft genome of *Steroidobacter cummioxidans* strain 35Y.**

| **Gene/Gene product** | **Locus** |
| --- | --- |
| Acyl-CoA synthetase | STC_00701, STC_01699, STC_03638, STC_04107, STC_04684, STC_04751, STC_04777, STC_05511, STC_05561, STC_06573, STC_06899 |
| Acyl-CoA-dehydrogenase | STC_00352, STC_00783, STC_01578, STC_01579, STC_01828, STC_02274, STC_03113, STC_03493, STC_04083, STC_04125, STC_04129, STC_04539, STC_04682, STC_04683, STC_04714, STC_05196, STC_05552, STC_05595, STC_06286 |
| Dienoyl-CoA reductase | STC_05558* |
| Enoyl-CoA hydratase/ isomerise | STC_00353, STC_00356, STC_02284, STC_02617, STC_03357, STC_04126, STC_04245, STC_04246, STC_04400, STC_04534, STC_04712, STC_05588, STC_05775, STC_05776, STC_05881 STC_06404 |
| 3-hydroxyacyl-CoA-dehydrogenase | STC_03203, STC_03640, STC_04527, STC_06353 |
| Acyl-CoA-acyltransferase/ ketoadipyl CoA thiolase | STC_03253 |
| α-Methylacyl-CoA racemase | STC_04710*, STC_05545* |

* implies the orthologous genes identified by comparative approach used in this study.

**Table G. Distribution and loci of methylcitrate pathway related putative genes in the draft genome of *Steroidobacter cummioxidans* strain 35Y.**

| **Gene/Gene product** | **Locus** |
| --- | --- |
| Methylcitrate synthase | STC_02290, STC_06452 |
| Methylcitrate dehydratase | STC_02291 |
| Methylaconitase | STC_05433*, STC_06403*, STC_06453* |
| Methylisocitrate lyase | STC_06451, STC_00216 |

* implies the orthologous genes identified by comparative approach used in this study.

**Table H. Distribution and loci of methylmalonyl-CoA pathway related putative genes in the draft genome of *Steroidobacter cummioxidans* strain 35Y.**

| **Gene/Gene product** | | **Locus** |
| --- | --- | --- |
| Propionyl-CoA carboxylase | α subunit | STC_02998* |
|  | β subunit | STC_02999* |
| Methylmalonyl-CoA epimerase | | STC_03217, STC_06893 |
| Methylmalonyl-CoA mutase | | STC_02273 |
| Succinyl-CoA synthetase | α subunit | STC_01268* |
|  | β subunit | STC_01269* |

* implies the orthologous genes identified by comparative approach used in this study.

**Table I. Distribution and loci of tricarboxylic acid cycle and glyoxylate bypass related putative genes in the draft genome of *Steroidobacter cummioxidans* strain 35Y.**

| **Gene/Gene product** | **Locus** |
| --- | --- |
| Citrate synthase | STC_00892,STC_04792, STC_05357 |
| Aconitate hydratase | STC_05433, STC_06403, STC_06453 |
| Isocitrate dehydrogenase | STC_03064, STC_03065 |
| 2-oxoglutarate dehydrogenase complex | STC_02410*, STC_04688, STC_05768, STC_06146, STC_06147, STC_06148 |
| Succinyl-CoA synthetase | STC_01268 |
| Succinate dehydrogenase | STC_06914, STC_06915, STC_06916, STC_06917 |
| Fumarate hydratase | STC_01694, STC_06399 |
| Malate dehydrogenase | STC_00782, STC_02242 |
| Malate synthase | STC_04389, STC_05847 |
| Isocitrate lyase | STC_04871, STC_05846 |

* implies the orthologous genes identified by comparative approach used in this study.

**Table J. Distribution and loci of gluconeogenesis related putative genes in the draft genome of *Steroidobacter cummioxidans* strain 35Y.**

| **Gene/Gene product** | **Locus** |
| --- | --- |
| Phosphoenolpyruvate carboxykinase (PEPCK) | STC_04427 |
| Phosphoenolpyruvate carboxylase | STC_02342 |
| Malic enzyme | STC_00798, STC_05319 |
| Phosphoenolpyruvate synthase (PEP synthase) | STC_06981 |

**Table K. Distribution and loci of polyhydroxyalkanoate and poly(3-hydroxybutyrate-co-3-hydroxyvalerate) biosynthesis related putative genes in the draft genome of *Steroidobacter cummioxidans* strain 35Y.**

| **Gene/Gene product** | **Locus** |
| --- | --- |
| PhaA (β-ketothiolase) [EC 2.3.1.9] | STC_00349, STC_00355, STC_ 00730, STC_00754, STC_02923, STC_04396, STC_04526, STC_04532, STC_04711, STC_05593, |
| PhaB (acetoacetyl-CoA reductase) [EC 1.1.1.36] | STC_03675* |
| PhaC (PHB synthase) | STC_01334 |
| Transcriptional regulator of phasin expression | STC_03342 |
| D-(-)-3-hydroxybutyrate oligomer hydrolase | STC_03891* |
| PHA depolymerase | - |
| Phasin phaP1/ phaP2/ phaP3 | - |

* implies the orthologous genes identified by comparative approach used in this study; (-) implies that genes are not detected.

**Table L. Distribution and loci of granulose biosynthesis related putative genes in the draft genome of *Steroidobacter cummioxidans* strain 35Y.**

| **Gene/Gene product** | **Locus** |
| --- | --- |
| ADP glucose pyrophosphorylase | STC_01339 |
| Granulose synthase | STC_01337 |

**Table M. Distribution and loci of cellulose biosynthesis related putative genes in the draft genome of *Steroidobacter cummioxidans* strain 35Y.**

| **Gene/Gene product** | **Locus** |
| --- | --- |
| Glucose kinase | STC_04707, STC_06330 |
| Phosphoglucomutase | STC_02859 |
| UDP-glucose pyrophosphorylase | STC_03312 |
| Cellulose synthase | STC_03100, STC_03514, STC_03680, STC_05743 |

**Table N. Distribution and loci of xanthan biosynthesis related putative genes in the draft genome of *Steroidobacter cummioxidans* strain 35Y.**

| **Gene** | **Locus** |
| --- | --- |
| gumB | STC_00998, STC_02100, STC_05657 |
| gumC | - |
| gumD | STC_04686, STC_00997, STC_02114, |
| gumE | - |
| gumF | - |
| gumG | - |
| gumH | - |
| gumI | STC_02864* |
| gumJ | STC_01009* |
| gumK | - |
| gumL | - |
| gumM | STC_04619* |

* implies the orthologous genes identified by comparative approach used in this study; (-) implies that genes are not detected.

**Table O. Distribution and loci of polyphosphates biosynthesis related putative genes in the draft genome of *Steroidobacter cummioxidans* strain 35Y.**

| **Gene/Gene product** | **Locus** |
| --- | --- |
| PPK1 (poly P kinase 1) | STC_02463, STC_03426, |
| PPK2 (poly P kinase 2) | STC_04761, STC_05227 |
| Polyphosphatase (PPX) | STC_03427 |

**Table P. Distribution and loci of xanthomonadin biosynthesis related putative genes in the draft genome of *Steroidobacter cummioxidans* strain 35Y.**

| **Gene/Gene product** | **Locus** |
| --- | --- |
| H (Halogenase) | STC_00698 |
| BP (Xanthomonadin biosynthesis protein) | STC_00699* |
| E (Xanthomonadin exporter) | STC_00700* |
| PSP (Putative secreted protein) | STC_00701* |
| BACPD (Xanthomonadin biosynthesis acyl carrier protein dehydratase) | STC_00703 |
| BA (Putative xanthomonadin biosynthesis acyltransferase) | STC_00702* |
| BMP (Putative xanthomonadin biosynthesis membrane protein) or Ketosynthase | STC_00704* |
| ACP (Acyl carrier protein) | STC_00686, STC_00695, STC_03115, STC_04247, STC_06362, STC_06878 |
| XanB1 (Putative reductase/halogenase) | STC_00689*, STC_00690* |
| XanB2 (Putative pteridine-dependent deoxygenase like protein) | STC_00694 |
| AMP-l (AMP-ligase) | STC_00693 |
| DP (Dipeptidyl peptidase) | STC_02322* |
| FabG (3-ketoacyl-ACP reductase) | STC_00691, STC_01873, STC_03105,  STC_04401, STC_04713, STC_06363,  STC_06534 |
| FabF (3-oxoacyl-ACP synthase) | STC_00692, STC_00696, STC_06361 |

* implies the orthologous genes identified by comparative approach used in this study.

**Table Q. Distribution and loci of putative urease genes in the draft genome of *Steroidobacter cummioxidans* strain 35Y.**

| **Gene/Gene product** | **Locus** |
| --- | --- |
| ureF; urease accessory protein | STC_04636 |
| ureE; urease accessory protein | STC_04637 |
| ureC; urease subunit alpha [EC:3.5.1.5] | STC_04638 |
| ureB; urease subunit beta [EC:3.5.1.5] | STC_04639 |
| ureA; urease subunit gamma [EC:3.5.1.5] | STC_04640 |
| ureD; urease accessory protein | STC_04641 |
| ureG; urease accessory protein | STC_04650 |
| ureJ; urease accessory protein | STC_04651 |

**Table R. Distribution and loci of urea transporter related putative genes in the draft genome of *Steroidobacter cummioxidans* strain 35Y.**

| **Gene/Gene product** | **Locus** |
| --- | --- |
| urtE; urea transport system ATP-binding protein | STC_04642 |
| urtD; urea transport system ATP-binding protein | STC_04643 |
| urtC; urea transport system permease protein | STC_04644 |
| urtB; urea transport system permease protein | STC_04645 |
| urtA; urea transport system substrate-binding protein | STC_04646 |

**Table S. Distribution and loci of putative polyketide synthase genes in the draft genome of *Steroidobacter cummioxidans* strain 35Y.**

| **Gene/ Gene Product** | **Locus** |
| --- | --- |
| Polyketide synthase | STC_04233, STC_04236, STC_05607, STC_05612 |

**Table T. List of putative carbohydrate metabolizing and transporting enzymes present in the draft genome of *Steroidobacter cummioxidans* strain 35Y.**

| **Sugar** | **Enzyme Family/ Domain** | **Locus** |
| --- | --- | --- |
| Arabinose | GH27 | STC_02600, STC_06772 |
|  | GH43 | STC_00236, STC_00372, STC_01198, STC_01860, STC_02911, STC_03625, STC_03629, STC_04722, STC_05696, STC_06266, STC_06465 |
|  | GH51 | STC_00231, STC_00241, STC_02908, STC_02912, STC_05833 |
| Rhamnogalacturonan | GH106 | STC_03649 |
|  | GH28 | STC_01425 |
| Xylan | GH10 | STC_03599 |
|  | CBM6 | STC_03851 |
| Mannose | GH1 | STC_01407 STC_05884 |
| Flucose | GH95 | STC_03802 |
| Pectin | PL1 | STC_01859, STC_01863, STC_02113, STC_05470, STC_06642 |
